# Molecular traits of MAPK kinases and the regulatory mechanism of *GhMAPKK5* alleviating drought/salt stress in cotton

**DOI:** 10.1093/plphys/kiae415

**Published:** 2024-08-14

**Authors:** Rui Ding, Junhua Li, Jie Wang, Yan Li, Wuwei Ye, Gentu Yan, Zujun Yin

**Affiliations:** Zhengzhou Research Base, State Key Laboratory of Cotton Bio-breeding and Integrated Utilization, School of Agricultural Sciences, Zhengzhou University, Zhengzhou 450001, China; Xinjiang Tarim River Seed Industry Co., Ltd., Xinjiang 518120, China; State Key Laboratory of Cotton Bio-breeding and Integrated Utilization, Institute of Cotton Research, Chinese Academy of Agricultural Sciences, Anyang 455000, China; Zhengzhou Research Base, State Key Laboratory of Cotton Bio-breeding and Integrated Utilization, School of Agricultural Sciences, Zhengzhou University, Zhengzhou 450001, China; State Key Laboratory of Cotton Bio-breeding and Integrated Utilization, Institute of Cotton Research, Chinese Academy of Agricultural Sciences, Anyang 455000, China; Western Agricultural Research Center, Chinese Academy of Agricultural Sciences, Changji 831100, China; Zhengzhou Research Base, State Key Laboratory of Cotton Bio-breeding and Integrated Utilization, School of Agricultural Sciences, Zhengzhou University, Zhengzhou 450001, China; State Key Laboratory of Cotton Bio-breeding and Integrated Utilization, Institute of Cotton Research, Chinese Academy of Agricultural Sciences, Anyang 455000, China; Western Agricultural Research Center, Chinese Academy of Agricultural Sciences, Changji 831100, China; Zhengzhou Research Base, State Key Laboratory of Cotton Bio-breeding and Integrated Utilization, School of Agricultural Sciences, Zhengzhou University, Zhengzhou 450001, China; State Key Laboratory of Cotton Bio-breeding and Integrated Utilization, Institute of Cotton Research, Chinese Academy of Agricultural Sciences, Anyang 455000, China; Western Agricultural Research Center, Chinese Academy of Agricultural Sciences, Changji 831100, China; Zhengzhou Research Base, State Key Laboratory of Cotton Bio-breeding and Integrated Utilization, School of Agricultural Sciences, Zhengzhou University, Zhengzhou 450001, China; State Key Laboratory of Cotton Bio-breeding and Integrated Utilization, Institute of Cotton Research, Chinese Academy of Agricultural Sciences, Anyang 455000, China; Western Agricultural Research Center, Chinese Academy of Agricultural Sciences, Changji 831100, China

## Abstract

Mitogen-activated protein kinase kinases (MAPKKs) play a critical role in the mitogen-activated protein kinase (MAPK) signaling pathway, transducing external stimuli into intracellular responses and enabling plant adaptation to environmental challenges. Most research has focused on the model plant Arabidopsis (*Arabidopsis thaliana*). The systematic analysis and characterization of *MAPKK* genes across different plant species, particularly in cotton (*Gossypium hirsutum*), are somewhat limited. Here, we identified *MAPKK* family members from 66 different species, which clustered into five different sub-groups, and *MAPKKs* from four cotton species clustered together. Through further bioinformatic and expression analyses, *GhMAPKK5* was identified as the most responsive *MAPKK* member to salt and drought stress among the 23 *MAPKKs* identified in *Gossypium hirsutum*. Silencing *GhMAPKK5* in cotton through virus-induced gene silencing (VIGS) led to quicker wilting under salt and drought conditions, while overexpressing *GhMAPKK5* in Arabidopsis enhanced root growth and seed germination under these stresses, demonstrating *GhMAPKK5*'s positive role in stress tolerance. Transcriptomics and Yeast-Two-Hybrid assays revealed a MAPK cascade signal module comprising GhMEKK (mitogen-activated protein kinase kinase kinases)3/8/31-GhMAPKK5-GhMAPK11/23. This signaling cascade may play a role in managing drought and salt stress by regulating transcription factor genes, such as *WRKYs*, which are involved in the biosynthesis and transport pathways of ABA, proline, and RALF. This study is highly important for further understanding the regulatory mechanism of *MAPKK* in cotton, contributing to its stress tolerance and offering potential in targets for genetic enhancement.

## Teaser

Genome-wide identification and transcriptomic analyses indicate that a mitogen-activated protein kinase kinase signaling cascade plays a role in managing drought and salt stress in cotton.

## Introduction

The effects of climate change have increasingly led to devastating environmental stresses, such as heat, salinity, and drought. All these environmental stressors are negatively impacting arable lands and plant production. Notably, drought and excessive salts are the most detrimental to reducing crop productivity. These stressors not only lower germination rates, but also detrimentally impact every stage of plant growth (Mareri et al. 2022). For mitigating the impact of these stresses, plants have evolved multiple molecular genetic mechanisms for recognizing the stresses, and then passing the signals to adjacent and distantly located plant cells/organs. Mitogen-activated protein kinase (MAPK) pathway involves a cascade of protein kinase activations and functions in central role in adapting to and surviving environmental challenges (Wang et al. 2020; Chen et al. 2021). In this pathway, plant senses environmental stimuli to activate MAP kinase kinase kinases (MAPKKKs or MEKKs), followed by triggering MAP kinase kinases (MAPKKs) and in turn further activating MAP kinases (MAPKs). Then, MAPKs can phosphorylate a range of substrates, including transcription factors, leading to changes in gene expression that enable the plant to respond to the initial signal (Zhang and Klessig 2001; Nolen et al. 2004). The MAPKK, as a key intermediate link of the MAPK cascade pathway, plays a key role in receiving upstream signals and activating downstream MAPK (Chen et al. 2021).

Plant *MAPKKs* are highly conserved (MAPK Group 2002). *MAPKKs* were first identified in the model plant Arabidopsis (*Arabidopsis thaliana*) (At), with a total of 10 *AtMAPKKs.* They are usually divided into four subfamilies. Three members of Group A MAPKKs, AtMAPKK1, AtMAPKK2, and AtMAPKK6, are associated with multiple abiotic stress response, such as wound, drought, cold, and salt-induced (Teige et al. 2004; Qiu et al. 2008; Guo et al. 2021). AtMAPKK3, a single member of group B, modulates light-mediated seedling development and root microtubule (Benhamman et al. 2017; Matilla 2022; Ojha et al. 2023), activated by AtMAPKKK18, and positively contributes to drought response via ABA-mediated stomatal closure (Li et al. 2017). Group C members, AtMAPKK4 and AtMAPKK5, modulate reactive oxygen species (ROS), regulate embryonic and stomatal development, and are involved in melatonin-mediated innate immune responses and auxin-mediated lateral root development via interaction with AtMAPK3/6 (Zhao et al. 2017; Liu et al. 2018; Thulasi Devendrakumar et al. 2018; Huang et al. 2019). In group D, the AtMAPKK9–AtMAPK3/6 cascade facilitates alternative respiration and sphingolipid activity in salt-stressed *Arabidopsis callus* (Liu et al. 2020; He et al. 2022; Chang et al. 2023; Li et al. 2023), while AtMAPKK7 and AtMAPKK10 working with AtMAPK3/6 trigger systemic immune response, shoot branching (Colcombet et al. 2016; Jia et al. 2016), and red light-regulated opening of seedling cotyledons (Xin et al. 2022). As such, it is obvious that MAPKK-mediated MAPK signaling cascade has been well characterized in the model plant Arabidopsis; the *MAPKK* family members have also been annotated in a number of plant species in recent years, including bread wheat (*Triticum aestivum* L.) (Zhan et al. 2017), rice (*Oryza sativa*) (Singh and Jwa 2013), potato (*Solanum tuberosum* L.) (Shang et al. 2022), and grapevine (*Vitis vinifera*) (Çakır and Kılıçkaya 2015). Nevertheless, only a few *MAPKKs* within these species have been characterized with broad roles in regulating grain yield (e.g. *OsMAPKK1*) (Chen et al. 2021), abiotic stress tolerance (e.g. *VvMAPKK2* and *VvMAPKK4*) (Wang et al. 2020), immune response (e.g. *StMAPKK5*) (Yang et al. 2023), and seed dormancy and germination (e.g. *TaMAPKK3*) (Martinez et al. 2020).

Similar to these species, our previous research has annotated the genomic-wide MAPKKs in cotton (*Gossypium hirsutum*); however, it lacks further characterization of any of these members (Yin et al. 2021). In this research, we extended the genomic annotation of MAPKKs from four commercially grown cotton species, covering *G. hirsutum*, *Gossypium barbadense*, *Gossypium arboretum*, and *Gossypium raimondii*, on the basis of which we used in silico analysis and RT-qPCR to identify an up-regulated *MAPKK* gene-*GhMAPKK5* (*Gh_A07G0124*) from *G. hirsutum* that likely is involved in stress tolerance in cotton. The genetic manipulation via virus-induced gene silencing (VIGS) in cotton and overexpression in Arabidopsis confirmed *GhMAPKK5*'s positive contribution to salt and drought tolerance. RNA sequencing analysis indicated that *GhMAPKK5* affects key component genes within abscisic acid (ABA) signaling, proline metabolism, and rapid alkalinization factor (RALF) synthesis and transport. These RNA-sequencing results were further backed up by supplying ABA, proline, and small peptides alleviating stress-sensitive phenotypes of VIGS cotton. In addition, a MAPK cascade signal module GhMEKK3/8/31–GhMAPKK5–GhMAPK11/23 was found. Our research is important for further understanding the regulatory mechanism of *MAPKK* in cotton in endowing stress tolerance.

## Results

### Phylogenetic analysis of *MAPKK* gene family

**Figure 1.**
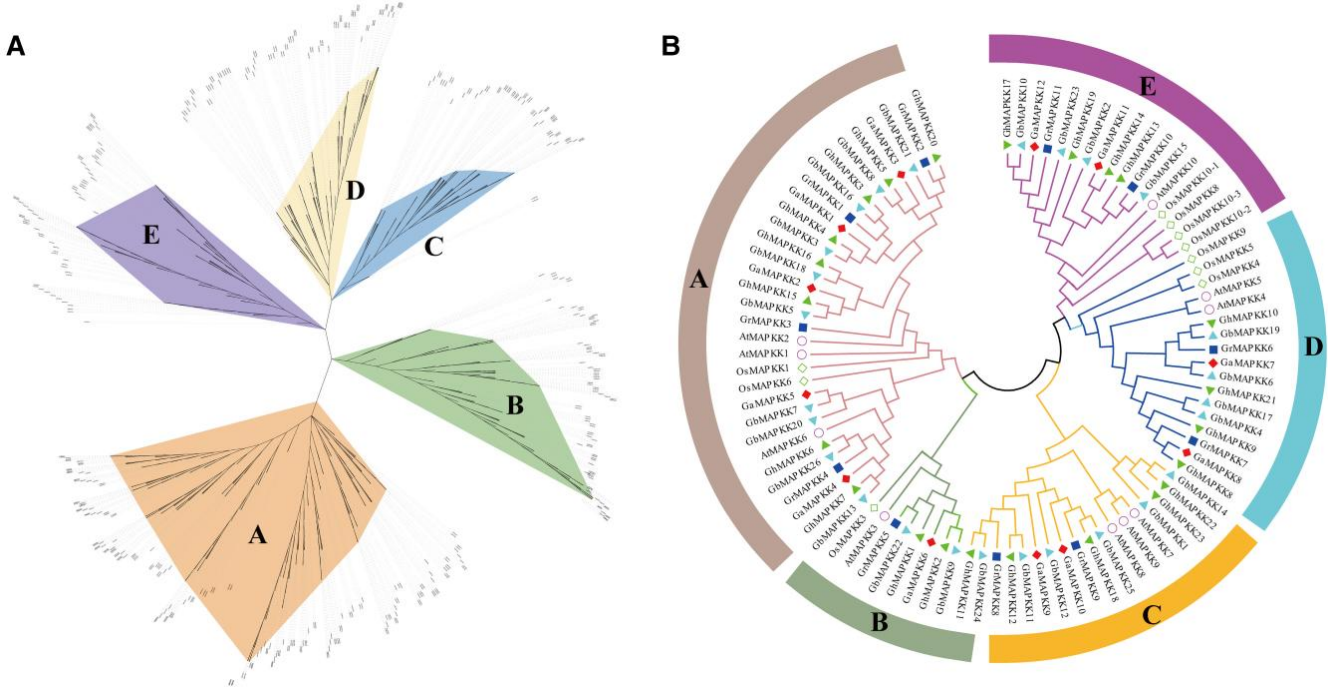
Phylogenetic analysis of MAPKK. **A)** Phylogenetic tree of MAPKKs among 66 species was established using MEGA7. **B)**  *MAPKK* phylogenetic analysis of Arabidopsis, rice, and four cotton species. Different colored shapes indicate different species genes, such as the solid green triangle representing *GhMAPKK*, which can also be seen from the gene ID next to it.

A phylogenetic tree was constructed of *MAPKKs* to clarify the evolutionary relationship amongst 66 different species (Fig. 1A). The number of *MAPKK* members in algae is small, such as *Klebsormidium nitens*, *Chlorella sorokiniana*, and *Gonium pectoral*, containing single member in each species. The number of *MAPKK* members seemly was not related to the genome size (Supplementary Table S1). For example, *Chlamydomonas incerta* has five *MAPKKs* in its 129.2 Mb genome, while *Capsicum annuum* has a similar number in its 3,480 Mb genome. As a tetraploid plant, *G. hirsutum* contains 23 *MAPKKs* equivalent to the sum of that in the two diploid ancestors, namely, 12 identified in *G. arboretum* and 11 in *G. raimondii* (Fig. 1B). Sequence alignment of cotton *MAPKK* proteins revealed that these proteins contained typical conserved motifs, S/T-X5-S/T (activation ring) and D (I/L/V) K motif (active site), which is similar to other species. The physicochemical properties of *MAPKK*-encoded proteins in four cotton species were predicted, including sequence length, molecular weight (*MW*), isoelectric point (*pI*), and subcellular location (Supplementary Table S2). According to sequence similarity (Supplementary Fig. S1), tree topology, the *MAPKKs* were classified into five clusters designated as A to E (Fig. 1). Group A had the largest number of *MAPKKs*, covering 186 members from all species. The *MAPKKs* from yeast and algae mainly fall in groups A and B, while *MAPKKs* from gymnosperms and angiosperms in Group C. Group D contained *MAPKKs* from mosses, ferns, land plants, and gymnosperms, but lacked members from monocot species. Interestingly, the members of the *MAPKKs* of the four cotton species are clustered together.

### Analysis of collinearity, *Ka*/*Ks* values, and chromosomal localization

**Figure 2.**
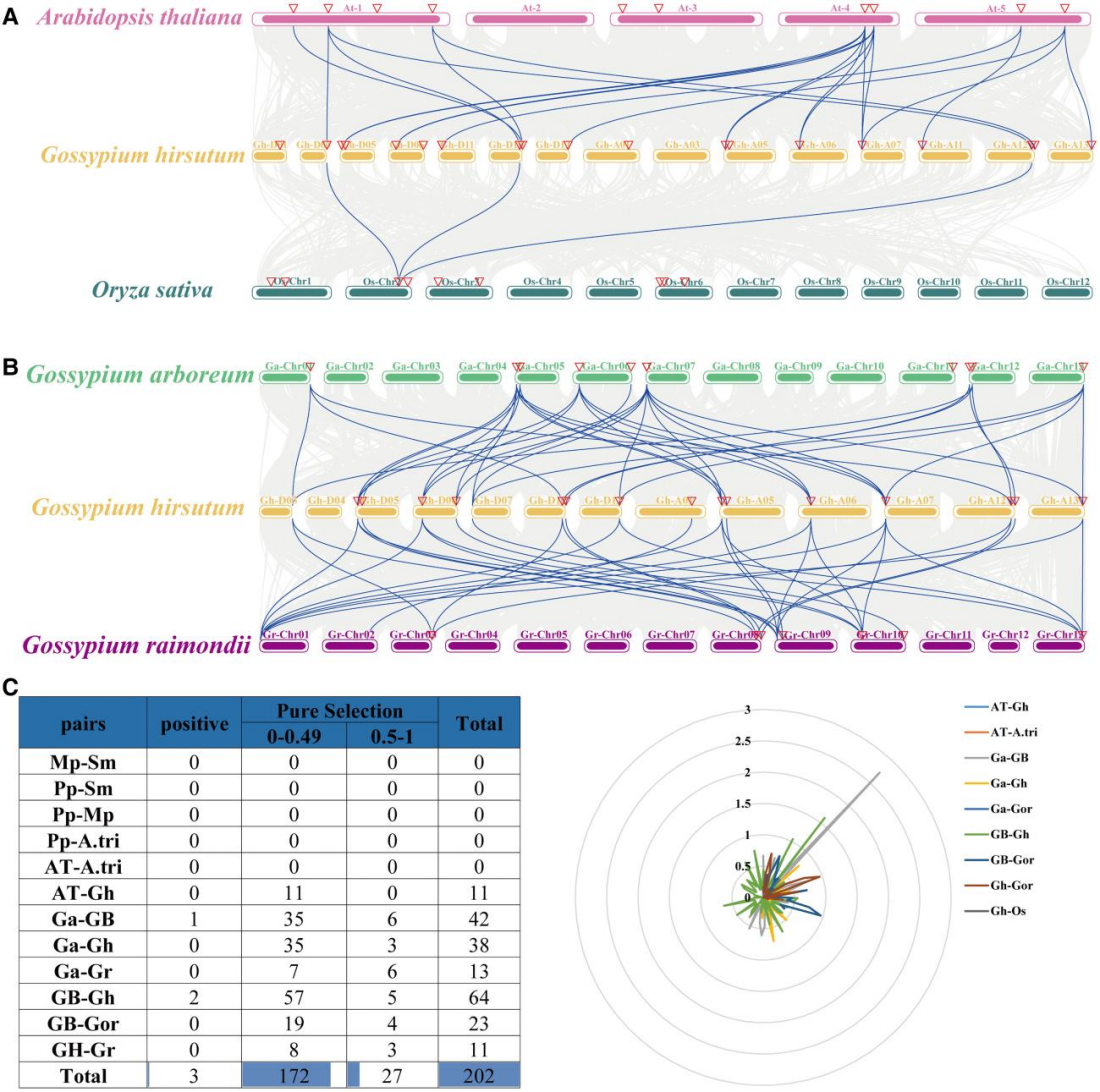
Collinearity analysis of *MAPKKs*. **A)** Collinearity analysis of *AtMAPKKs*, *OsMAPKKs*, and *GhMAPKKs*. **B)** Collinearity analysis of *GaMAPKKs*, *GhMAPKKs*, and *GrMAPKKs*. **C)** Repeated gene pairs and *Ka*/*Ks* ratio of different species were analyzed, and *Ka*/*Ks* radar map was obtained.

Seven *AtMAPKK* genes showed collinearity with 14 *GhMAPKK* genes, and three *OsMAPKK* genes were homologous to three *GhMAPKK* genes (Fig. 2A). *G.hirsutum* displayed 35 and 33 homoeologous gene pairs with *G. arboreum* and *G. raimondii*, indicating collinearity across multiple *GaMAPKK* and *GrMAPKK* genes (Fig. 2B). The *GhMAPKK* genes were found collinear with multiple *GaMAPKK* and *GrMAPKK* genes; the number of *MAPKK* genes in cotton was amplified mainly due to genome-wide replication and fragment duplication. Analysis using the *Ka/Ks* ratio to explore *MAPKK* gene evolution post-polyploidy showed most genes with the *Ka*/*Ks* < 1, suggesting conservation. However, three duplicated gene pairs had *Ka*/*Ks* > 1, showing that they had evolved more rapidly and were under positive selection possibly due to adaptive changes post-duplication (Fig. 2C).

Chromosome distribution maps for the *MAPKK* gene family in four cotton species were created, showing genetic divergence and duplication. Of the 72 *MAPKK* genes identified across these species, 69 were chromosomally mapped, while three were on unmapped scaffolds. The distribution of *MAPKK* of cotton on chromosome is not uniform. In *G. hirsutum*, *G. arboreum*, and *G. barbadense*, *MAPKK* genes were more abundantly found on chromosome 5 (Chr05) and Chr07. In *G. raimondii*, these genes were mostly distributed on Chr08 and Chr09 (Supplementary Fig. S2).

### Analysis of gene structure, conserved motifs, and *cis*-acting elements

The evolution of the *MAPKK* gene family was investigated through gene structure and conserved motifs. *MAPKK* genes in each subgroup had similar exon-intron arrangement. Subgroups A and B contained seven to eight introns, while the genes of the other subgroups were mostly intron-free (Supplementary Fig. S3, A and D). All *MAPKK* members had conserved motifs 1, 2, and 8, and the motif arrangement in the same clade was similar (Supplementary Fig. S3, A and B). The different composition of motifs also explained the functional diversity of genes during evolution. Most MAPKK proteins contain Pkc_MAPKK_plant_like (cd06623) domains that have protein kinase activity (Supplementary Fig. S3, A and C).

**Figure 3.**
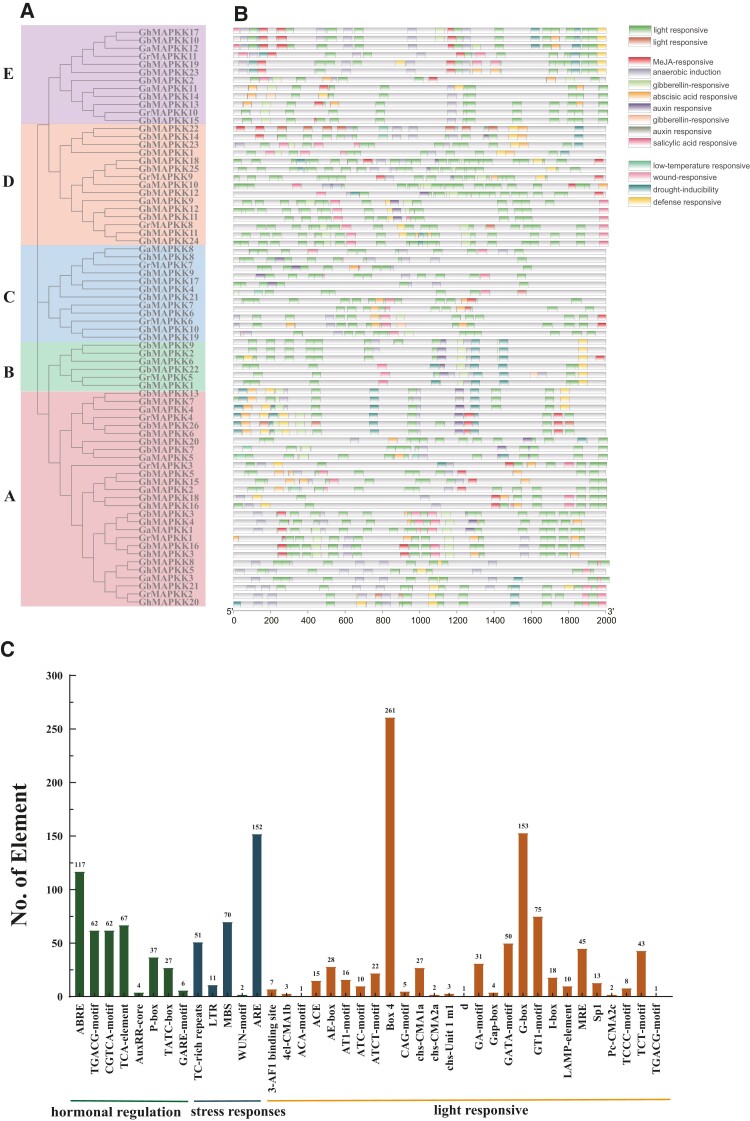
Analysis of *cis*-acting element numbers of *MAPKKs* in four cotton species. **A)** Phylogenetic tree of MAPKKs among four cotton species. **B)** The color of the grid represents the number of *cis*-acting elements of the *MAPKK*s. **C)** Statistical classification of the number of components.

To determine the potential of *MAPKK*s in drought tolerance and salt tolerance, the putative stress-related and plant hormone-related *cis*-acting elements were analyzed and grouped into three functional types, as shown in Fig. 3. The distribution of these elements varied across subgroups, with groups A and D containing the highest abundance. Some genes in groups A and E exhibited elements related to ABA (ABRE), auxin (AuxRR-core), defense and stress (TC-rich repeats, MBS), gibberellin (P-box, TATC-box and GARE-motif), and MeJA (TGACG-motif and CGTCA-motif), suggesting their regulatory role in hormonal or stress responses. Group C lacks defense (TC-rich repeats), drought (MBS), and other stress-related elements, and Group B lacks elements associated with ABA and low temperature response (Fig. 5, A and B). Light-responsive elements (854) were most abundant, particularly Box 4 (261), G-box (153), and tc (Fig. 3, B and C).

### Expression pattern of *MAPKK* genes and their response to abiotic stresses

**Figure 4.**
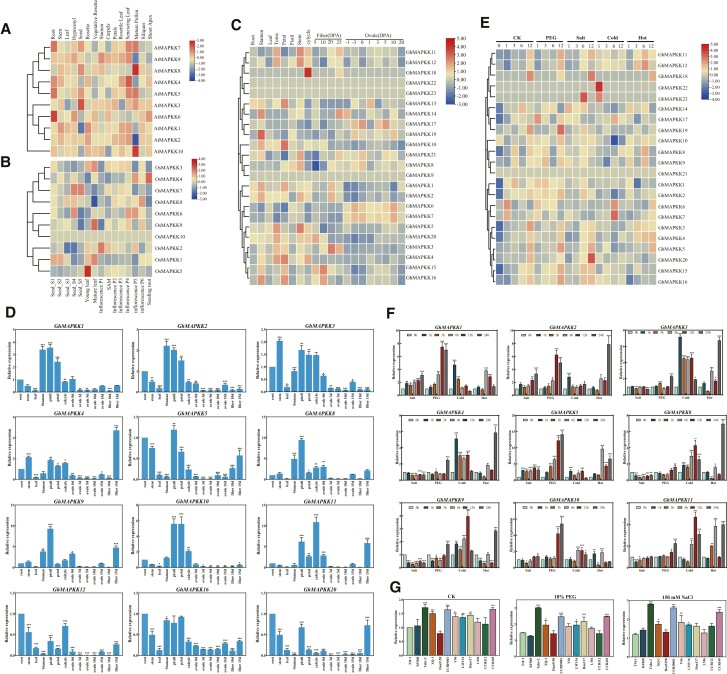
Expression patterns of *MAPKKs*. **A)** Tissue/organ-specific expression heat maps of *AtMAPKKs* in Arabidopsis. **B)** Tissue/organ expression heat maps of *OsMAPKKs* in rice. **C)** Tissue/organ-specific expression heat maps of *GhMAPKKs* in cotton. **D)** Relative expression levels of 12 *GhMAPKK*s in cotton. **E)** Stress-induced expression patterns of *GhMAPKKs* under PEG, salt, heat and cold treatments for 1, 3, 6, 12, and 24 h. **F)** Relative expression level of *GhMAPKKs* after exposing to different stresses. **G)** Expression of *GhMAPKK5* in different cotton varieties. Three biological replicates were performed for each experiment, with 15 plants per replicate. One-way analysis of variance was used. The error bars represent mean ± SD of at least three biological replicates (*P < 0.05, **P < 0.01, ***P < 0.001). PEG (polyethylene glycol).

The expression patterns of the *MAPKK* gene family members in various tissues of Arabidopsis, *O. sativa* and *G. hirsutum*, were studied using RNA-seq data to elucidate their functions. In Arabidopsis, all 10 *AtMAPKK* genes were all detectable across tissue, with notable expression patterns: *AtMAPKK3* was highly expressed in seeds; *AtMAPKK6* was highly expressed in root; *AtMAPKK4* was highly expressed in leaves; and *AtMAPKK8*/*10* was highly expressed in mature pollen (Fig. 4A). In *O. sativa*, *OsMAPKK10* was undetectable in all tissues, while *OsMAPKK1* and *OsMAPKK7* were highly expressed in seeds, *OsMAPKK5* in leaves, and *OsMAPKK4*/*6* in inflorescence (Fig. 4B). The expression of *GhMAPKKs* showed to be differentially expressed in the whole plant, except *GhMAPKK18/22/23.* The expression of other 20 genes was relatively high in all tissues, including significant expression in stamen, torus, petals, stems, and ovules (Fig. 4C). To validate the RNA-seq findings, 12 *GhMAPKKs* were randomly selected for RT-qPCR, which confirmed the RNA-seq expression profiles, highlighting the diverse and tissue-specific expression patterns of the *MAPKK* gene family (Fig. 4D).

We further examined the regulation *of MAPKK* genes from Arabidopsis, rice, and cotton by stress. Several *AtMAPKK* genes (*AtMAPKK1/2/6/4/5/7/9*) were upregulated by drought, with *AtMAPKK6/4/5/7/9* significantly upregulated at early stage of treatment for 1 h, but downregulated later, while *AtMAPKK1/2* maintained an upregulated trend. Under salt stress, all other *AtMAPKK*s were upregulated, except *AtMAPKK6/8*. Cold stress only significantly increased *AtMAPKK7/8/9* expression. High temperatures reduced decreased *AtMAPKKK* expression, with a few exceptions (Supplementary Fig. S4A). These patterns suggest that *AtMAPKKs* play crucial roles in Arabidopsis' response to drought and salt stress, corroborated by RT-qPCR data (Supplementary Fig. S4A). RT-qPCR data showed that the expression of most *OsMAPKK* genes in rice was downregulated under drought and salt treatment. However, *OsMAPKK6* and *OsMAPKK10-2* were upregulated under salt treatment, and *OsMAPKK8/9/10-1/10-2* showed significant upregulation after drought treatment (Supplementary Fig. S4B).

In *G. hirsutum*, 23 *GhMAPKKs* showed varied responses to stress (Fig. 4E). Drought stress predominantly upregulated Group A *GhMAPKKs*. Salt treatment increased the expression level of *GhMAPKK5* (Group A) and *GhMAPKK8/22/23* (Group D), along with downregulation or unchanged expression of several Group C *GhMAPKK* genes. Cold stress specifically enhanced *GhMAPKK6/7* expression, with an initial increase in *GhMAPKK22/23* expression, followed by a decrease. Excessive heat caused an upregulation in only *GhMAPKK3-6*. These findings indicate both functional redundancy and differentiation within the *GhMAPKK* subfamilies. It is noted that *GhMAPKK5* was stood out for its significantly higher expression and consistent upregulation following drought and salt stress (Fig. 4F), highlighting its potential key role in cotton's stress adaptation. Under drought and salt stress, the expression of *GhMAPKK5* in Tahe-2 was higher than that of other varieties (Fig. 4G).

### Virus-induced gene silencing (VIGS) of *GhMAPKK5* reduces drought and salt tolerance in cotton plants

**Figure 5.**
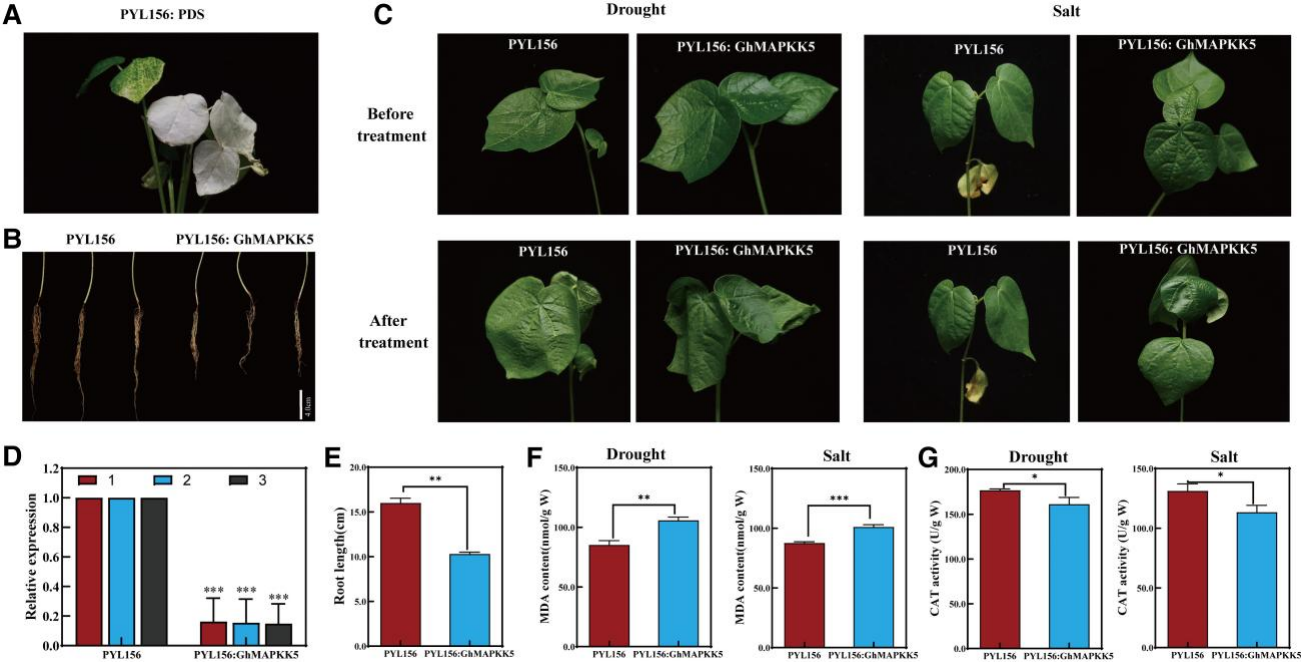
Silencing of *GhMAPKK5* compromised cotton drought and salt stress tolerance. **A)** Albino marker plant. **B)** Comparison of root length between PYL156: *GhMAPKK5* silenced plants and PYL156 plants. **C)** Comparison of phenotypes of VIGS gene-silenced plants and control plants with pYL156 under drought or salt treatment. **D)** Relative silencing efficiency, VIGS, and control plants analyzed by qRT-PCR. **E)** PYL156: *GhMAPKK5* gene-silenced plant root length measurement. **F)** Determination of MDA content under drought and salt stress. **G)** Determination of CAT activity under drought stress and salt stress. Three biological replicates were performed for each experiment, with 15 seedlings in each replicate. One-way analysis of variance was used. The error bars represent mean ± SD of at least three biological replicates (*P < 0.05, **P < 0.01, ***P < 0.001). MDA (malondialdehyde); CAT (catalase).

Cotton plants infected with pYL156: PDS vector showed albino phenotype. The RT-qPCR analysis was performed to confirm successful *GhMAPKK5* silencing by VIGS (Fig. 5, A and D). It was found that the root length of PYL156: *GhMAPKK5* was shorter than that of the control (Fig. 5, B and E). Following 150 mM NaCl and 18% PEG (to mimic drought) treatment for 6 h, VIGS gene-silenced cottons were subjected to significant wilting, compared to control seedlings (Fig. 5C). The contents of malondialdehyde (MDA) and catalase (CAT) activity were closely related to stress injury. Under simulated drought and salt stress, the MDA content of PYL156: *GhMAPKK5* plants was 105.82 nmol/g W, which is much higher than that of control plants (Fig. 5F). The activity of CAT was significantly lower than that of control plants (Fig. 5G). This outcome indicated that *GhMAPKK5* positively contributes to cottons' stress tolerance, such as drought and salt.

### Differentially expressed genes (DEGs), GO analysis, and KEGG enrichment in transcriptome data

**Figure 6.**
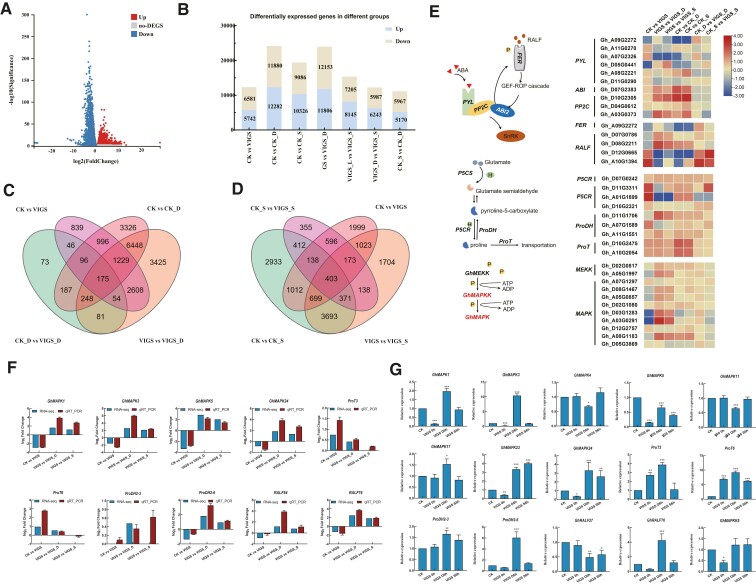
Details of RNA-seq data differentially expressed genes (DEGs) in each comparison set. **A)** Volcano plot, identification of DEGs by Log2FC, and q-value (FDR) ≤ 0.05. Blue-colored dots indicate downregulation of genes, while red-colored dots indicate upregulation of genes. **B)** Bar graph representing four combinations; total, blue indicates the number of up-regulated genes, and yellow color denoting the number of down-regulated genes. **C)** Venn diagram showing overlapping of total DEGs among four comparison sets under drought stress. **D)** Venn diagram showing overlapping of total DEGs among four comparison sets under salt stress. **E)** DEGs in ABA signaling pathway, *MAPK* signaling pathway, and synthesis and transportation of proline. **F)** Ten selected genes were examined for their expression in response to drought and salt stress. The expression levels of stress-treated samples are indicated by the log2 fold change relative to that of the corresponding control samples. The means were taken from three independent replications. **G)** The expression of 15 signaling pathway genes under drought and salt stress was studied. Three biological replicates were performed for each experiment, with 15 plants per replicate. A *t*-test was used for statistical analysis. The error bars represent mean ± SD of at least three biological replicates (*P < 0.05, **P < 0.01, ***P < 0.001). CK = pYL156; CK_D = pYL156 drought 6 h; CK_S = pYL156 salt 6 h; VIGS = pYL156: *GhMAPKK5*; VIGS_D = pYL156: *GhMAPKK5* drought 6 h; and VIGS_S = pYL156: *GhMAPKK*5 salt 6 h. DEGs (different gene expression); ABA (abscisic acid); PYL (Pyrabactin Resistance/Pyrabactin Resistance-Like/Regulatory Component of ABA receptors); PP2C (Protein Phosphatase 2C); ABI2 (abl interactor 2); RALF (rapid alkalinization factor); FER (FERONIA, belongs to the CRRLK1L-like receptor protein kinase family); GEF-ROP cascade (guanine nucleotide exchange factors–Rho of plants cascade); SnRK (SNF-related serine/threonine-protein kinase); P5CS (pyrroline-5-carboxylate synthetase); P5CR (pyrroline-5-carboxylate reductase); ProDH (proline dehydrogenase); ProT (proline transporter); MEKK (mitogen-activated protein kinase kinase kinase); MAPKK (mitogen-activated protein kinase kinase); MAPK (mitogen-activated protein kinase); ADP (adenosine diphosphate); ATP (adenosine triphosphate).

Following the observation of *GhMAPKK5* silencing cotton's phenotype, we performed RNA-seq analysis to provide insights into the role of *GhMAPKK5* in cotton under various stress conditions. High quality of the sequencing data, with a total of 114.83 Gb clean reads, was obtained from the extracted RNA, as indicated by the high Q20 (97.53%) and Q30 (92%) base percentages and efficient mapping range from 95.38% to 96.86%, laying a solid foundation for reliable downstream analyses (Supplementary Table S3). Pearson correlation coefficient revealed high consistency within the 18 library bioreplicates with repetition values above 94% and the correlation coefficients among different samples below 90%, highlighting distinct differences among them (Supplementary Fig. S5). The volcano map showed blue dots for downregulated genes and red dots for upregulated genes, while gray dots marked genes without differential expression (Fig. 6A). The comparison between control (CK) and virus-induced gene silencing (VIGS) plants revealed more than 12,000 DEGs, indicating a broad regulatory role for *GhMAPKK5* in cotton's response to environmental stresses (Fig. 6B). Besides, over 6000 DEGs were covered in both VIGS *vs* VIGS_D and CK *vs* CK_D comparisons, although around 3000 DEGs unique in CK *vs* CK_D (Fig. 6C). Under salt conditions, 5,166 genes were overlapped between VIGS *vs* VIGS_S in comparison to CK *vs* CK_S, where 3038 DEGs were unique in VIGS *vs* VIGS_S (Fig. 6D). Further analysis of DEGs under stress conditions allowed us to categorize them into three main groups: biological processes, cell components, and molecular functions. Gene ontology (GO) terms associated with kinase activity, transport and DNA replication were highly enriched in the comparison between CK and VIGS. Under drought and salt stress, the biological processes were mainly enriched in regulation of transcription, phosphorus metabolic process, protein phosphorylation, carboxylic acid metabolic process, and organic acid metabolic process (Supplementary Fig. S6). GO enrichment analysis brought to light a myriad of differentially expressed transcription factors (TFs) vital for drought and salt stress responses, which were categorized into several families, including *WRKY*, MYB (Myeloblastosis), AP2-EREBP (APETALA2/ethylene-responsive element binding protein), bZIP (basic region-leucine zipper), and bHLH (basic helix–loop–helix) (Supplementary Table S6 and Supplementary Table S4 and Supplementary Table S5).

The study also extended into the Kyoto Encyclopedia of Genes and Genomes (KEGG) pathway analysis, which further substantiated the involvement of *GhMAPKK5* in stress responses of 12, 27, and 23 KEGG pathways under control, drought, and salt conditions, respectively (Supplementary Fig. S7). These functional annotations of DEGs revealed significant enrichment in pathways associated with plant–pathogen interaction, MAPK signaling pathway–plant, plant hormone signal transduction, and carbon fixation in photosynthetic organisms, alongside secondary metabolic pathways crucial under drought stress, such as carotenoid biosynthesis, biosynthesis of cofactors, and vitamin B6 metabolism, primary and central metabolic pathways under salt stress, such as biosynthesis of cofactors, ascorbate, and aldarate metabolism, and zeatin biosynthesis (Supplementary Fig. S7).

Additionally, the functional annotations also highlighted DEGs related to MAPK signal transduction, hormone signaling pathway, and amino acid metabolism; therefore, we further explored the expression changes of genes involved in the biosynthesis of phytohormones, such as ABA, proline, and RALF (Fig. 6E). The ABA signal transduction pathway, for example, revealed specific gene families *Pyrabactin Resistance/Pyrabactin resistance-like/Regulatory Component of ABA Receptors* (*PYR/PYL/RCAR*), and *Protein Phosphatase 2C* (*PP2C*) downregulated by *GhMAPKK5* silencing in both control and stress conditions. Two *RALF* synthesizing genes were upregulated in VIGS and controls, but the receptor gene *FER* was downregulated. Moreover, *ABI2* in the pathway of small peptide action can activate *PP2C* in the ABA pathway, and the two pathways influence each other (Fig. 6E and Supplementary Table S6). Furthermore, the proline synthesis and transport processes were examined, identifying upregulation in most gene members of *Pyrroline-5-Carboxylate Reductase* (*P5CR*), *Pyrroline-5-Carboxylate Synthetase* (*P5CS*), *Proline Dehydrogenase* (*ProDH*), and Proline Transporter (*ProT*) families, indicative of their stress response activation. In *MAPK* signaling pathway, five genes were downregulated after VIGS treatment, among which, three were *MAPK4* homologous genes (*Gh_D08G1467*, *Gh_A05G0857*, and *Gh_A08G1183*), and two were *MAPK3* homologous genes (*Gh_D03G1283* and *Gh_A03G0291*). We performed RT-qPCR analysis using the same samples to verify our RNA-Seq findings. Using correlation analysis, the fold change (FC) of genes between tissues subjected to drought/salt and control treatments was compared in RT-qPCR *vs.* RNA-Seq (Fig. 6F). The relative expression pattern in RT-qPCR was consistent with that observed in RNA-Seq data, albeit minor differences in absolute expression level (Fig. 6G).

### Supplying ABA, proline, and RALF alleviated the drought- and salt-sensitive phenotypes of *GhMAPKK5* silenced plants

**Figure 7.**
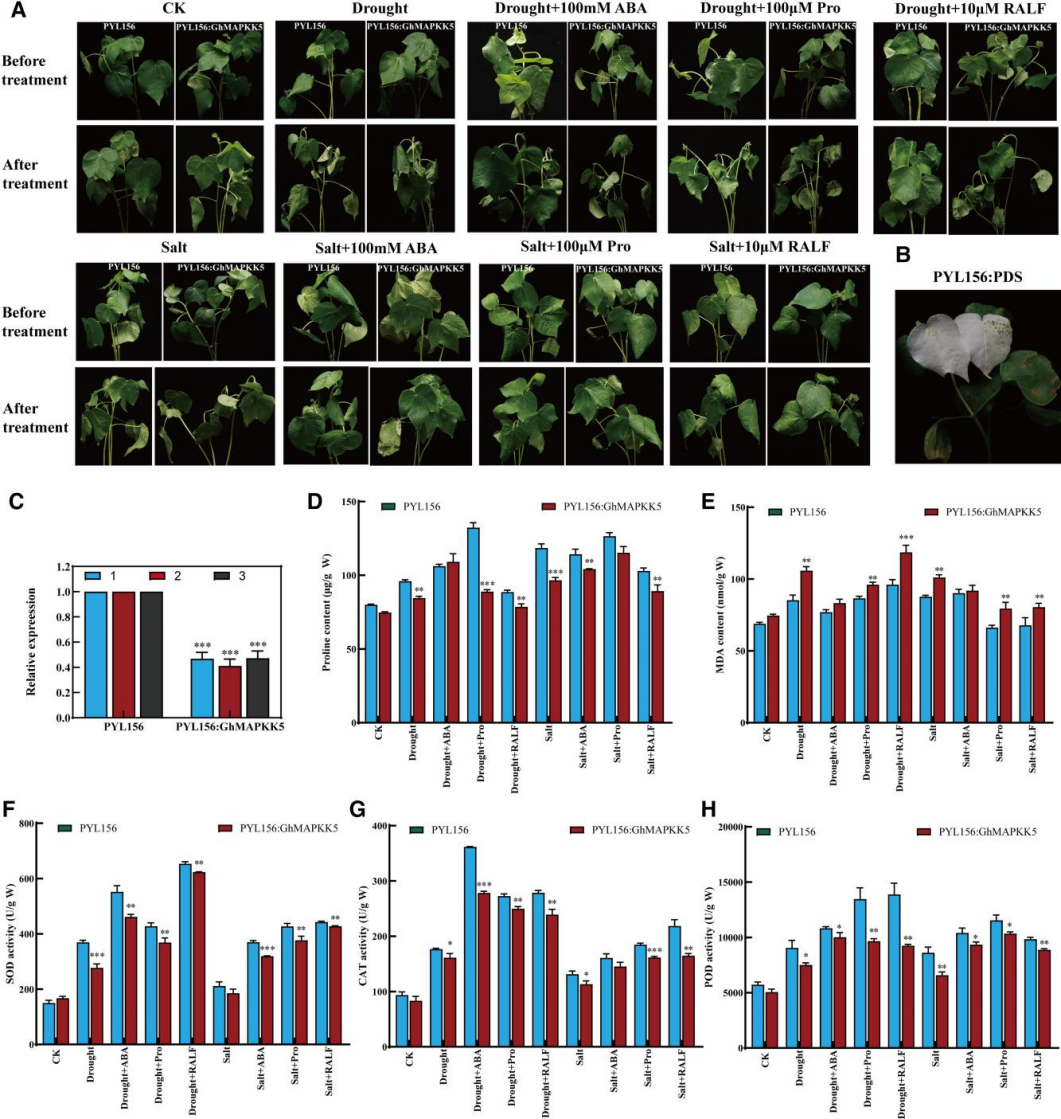
Comparison of phenotypes and estimation physiological indexes before and after treatment of exogenous substances. **A)** Comparison of phenotypes before and after treatment. **B)** Albino marker plant. **C)** Relative silencing efficiency, VIGS, and control plants analyzed by RT-qPCR. VIGS expression was declined to less than half of the control plants. **D)** Proline content. **E)** MDA content. **F)** SOD activity. **G)** CAT activity. **H)** POD activity. A *t*-test was used for statistical analysis. Three biological replicates were performed for each experiment, with 15 plants per replicate. The error bars represent mean ± SD of at least three biological replicates (*P < 0.05, **P < 0.01, ***P < 0.001). ABA (abscisic acid); Pro (proline); RALF (rapid alkalinization factor); MDA (malondialdehyde); CAT (catalase); SOD (superoxide dismutase); POD (peroxidase).

The transcriptomics data hinted at the involvement of GhMAPKK5*-*mediated MAPK cascade probably involved in ABA signaling pathway, RALF synthesis and transport, and proline synthesis and transport. Therefore, in order to verify our hypothesis, *GhMAPKK5* gene-silenced plants were treated with exogenous ABA, proline, and RALF small peptide spray, and the phenotypes of the plants were observed, and related physiological indicators were detected. The bleaching of PYL156: PDS plants and the reduction of *GhMAPKK5* expression in PYL156: *GhMAPKK5* plants by 50% suggested that *GhMAPKK5* was successfully silenced in cotton plants, and the plants could be used for subsequent treatment (Fig. 7, B and C). When exposed to 150 mM NaCl and 18% PEG, *GhMAPKK5*-silenced cottons exhibited significant wilting of the seedlings. However, spraying the leaves with ABA, proline, and RALF peptides notably enhanced the resilience of *GhMAPKK5*-silenced seedlings, as observed in the improvements in their phenotypes (Fig. 7A). Under PEG treatments (to mimic drought stress), the levels of peroxidase (POD), superoxide dismutase (SOD), catalase (CAT), and proline contents in *GhMAPKK5*-silenced plants were lower as compared to control plants, whereas malondialdehyde (MDA) content was significantly higher in *GhMAPKK5*-silenced plants, indicating greater stress damage. A similar pattern was also observed under salt stress. Interestingly, the negative effects of both drought and salt stress were alleviated by the application of exogenous ABA, proline, and small peptide treatments, which decreased MDA accumulation and increased the activities of POD, SOD, and CAT as well as proline productions, highlighting an enhanced stress tolerance by the supplements (Fig. 7, A and D to H).

### Overexpressing *GhMAPKK5* improved stress tolerance of Arabidopsis

**Figure 8.**
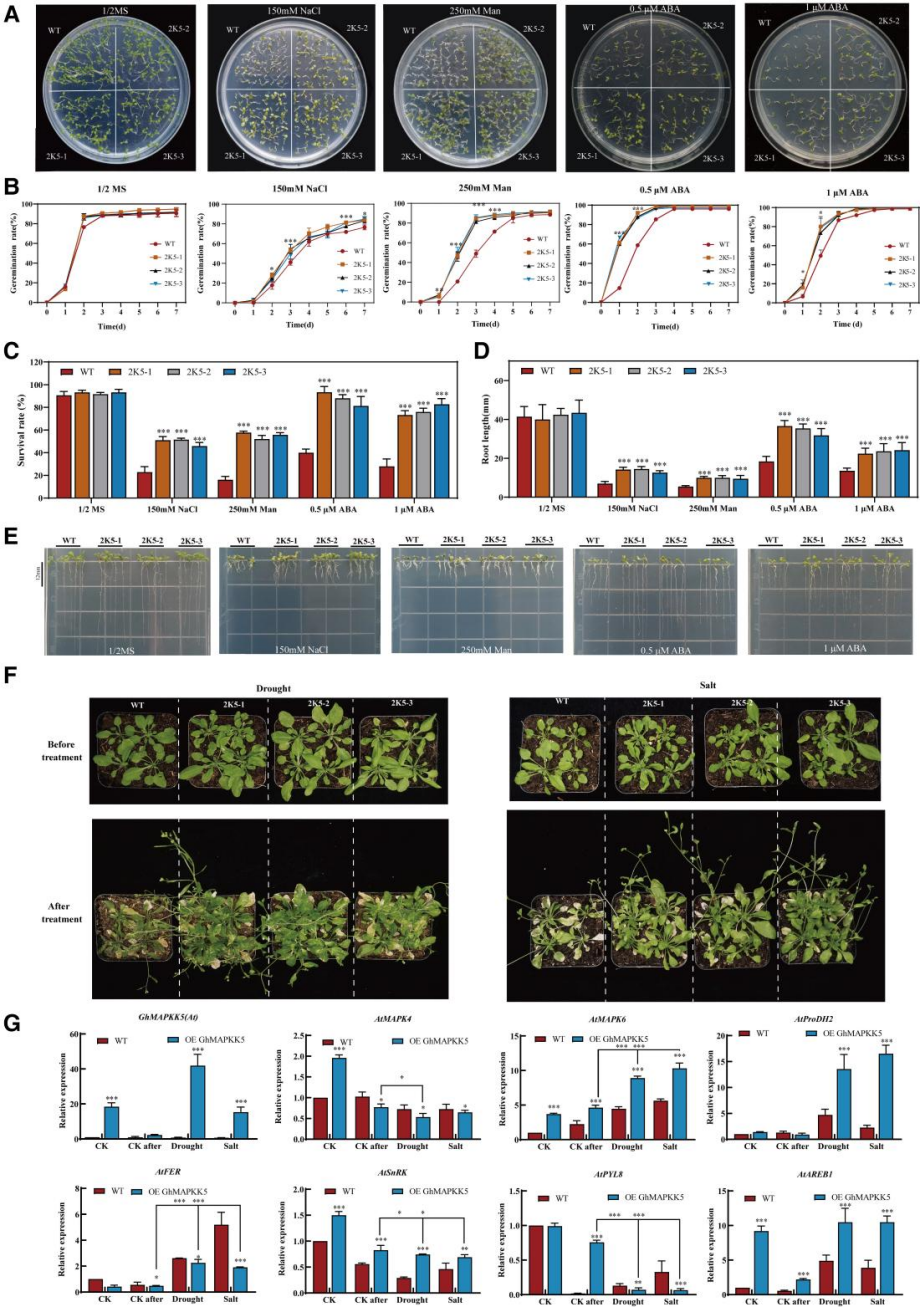
Overexpression of *GhMAPKK5* improved stress tolerance of Arabidopsis. **A, B)** Germination of transgenic and wild-type seed of Arabidopsis sown on 1/2 MS medium supplemented with 150 mM NaCl, 250 mM Man, 0.5 μM ABA, and 1 μM ABA. The 1/2 MS medium without any treatment was used as the control. **C)** Survival rate of transgenic seeds and WT treated with 150 mM NaCl, 250 mM Man, 0.5 μM ABA, and 1 μM ABA. **D, E)** Comparison of root length between transgenic plants and WT grown on 1/2 MS supplemented with 250 mM Man, 150 mM NaCl, 0.5 μM ABA, and 1 μM ABA for 10 days. **F)** Transgenic lines and WT under drought and saline conditions. **G)** Detection of related gene expression. One-way analysis of variance was used. The error bars represent ± SD of at least three biological replicates (*P < 0.05, **P < 0.01, ***P < 0.001). Man (mannitol); ABA (abscisic acid); WT (wild type); MAPKK (mitogen-activated protein kinase kinase); MAPK (mitogen-activated protein kinase); ProDH (proline dehydrogenase); FER (FERONIA, belongs to the CRRLK1L-like receptor protein kinase family); SnRK (SNF-related serine/threonine–protein kinase); PYL (pyrabactin resistance/pyrabactin resistance-like/regulatory component of ABA rrceptors); ABRE (ABA responsive element).

Given that *GhMAPKK5*'s significant roles in stress tolerance in cotton, we engineered this gene in Arabidopsis and attempted to improve its stress resilience. Three lines overexpressing *GhMAPKK5* (2K5-1, 2K5-2, 2K5-3), were selected and their root growth, germination rate, and survival rate on 1/2 MS medium were studied to explore their drought tolerance, salt tolerance, and response to ABA. The overexpressing *GhMAPKK5* lines (2K5-1, 2K5-2, 2K5-3) showed better growth under 150 mM NaCl, 250 mM mannitol, and ABA treatments, respectively, compared to wild-type (WT) seedlings (Fig. 8A). There was no penalty of germination and survival rates of transgenic seedlings under normal conditions (Fig. 8, A to C). Salt treatment delayed the germination rate in all plants, with the overexpression lines showing a slightly higher germination rate at 7 days. Under 250 mM mannitol and ABA condition, the germination rate of overexpressed plants was still higher than that of WT plants during initial 4 days, although rates equalized after 7 days (Fig. 8B).

Post-germination, continuous salt, mannitol, and ABA treatments adversely affected the survival of all plants, yet the overexpression lines maintained a notably higher survival rate than WT plants (Fig. 8, B and C). The root growth was similar between overexpression and WT plants, not different from that of WT under normal conditions. Both experienced significant inhibition under salt, osmotic, and ABA stress; however, the overexpressing lines exhibited longer root lengths than WT under these stressful conditions, indicating enhanced tolerance of engineered plants. With the increase of ABA concentration, the degree of root growth inhibition was enhanced (Fig. 8, D and E).

In addition, we added exogenous substances proline, RALF, and ABA to 150 mM NaCl and 250 mM mannitol 1/2 MS medium and also found that the addition of exogenous substances can improve the germination rate, survival rate, and root growth of Arabidopsis seeds under stress conditions. These foreign substances may be related to the drought and salt tolerance mechanism regulated by *GhMAPKK5* (Supplementary Figs. S8 and S9).

As the osmotic stress by mannitol supplement is a key impact by water-deficit stress in soil, we investigated whether these overexpression lines not only confer tolerance to osmotic stress, but also drought tolerance in soil. We further examined the phenotypes of these plants under salt and drought treatment in the soil. Four-week-old transgenic plants and WT plants were subjected to saline water (150 mM NaCl) and drought stress (withholding water) in soil. After 14 days post treatment, WT plants showed reduced growth, wilting, and less bolting, indicative of stress impact. In contrast, the overexpressing plants maintained larger rosettes, lush and green foliage with fewer dead leaves, and more bolting (Fig. 8F), signifying that *GhMAPKK5* overexpression conferred enhanced tolerance to both salt and drought tolerance in Arabidopsis plants. Furthermore, the expression levels of some genes of Arabidopsis were detected by sampling after treatment. The gene expression levels of *MAPK4*, *MAPK6*, *ProDH*, *FER*, and *PYL* were significantly changed (Fig. 8G).

### Interaction analysis of GhMAPKK5

**Figure 9.**
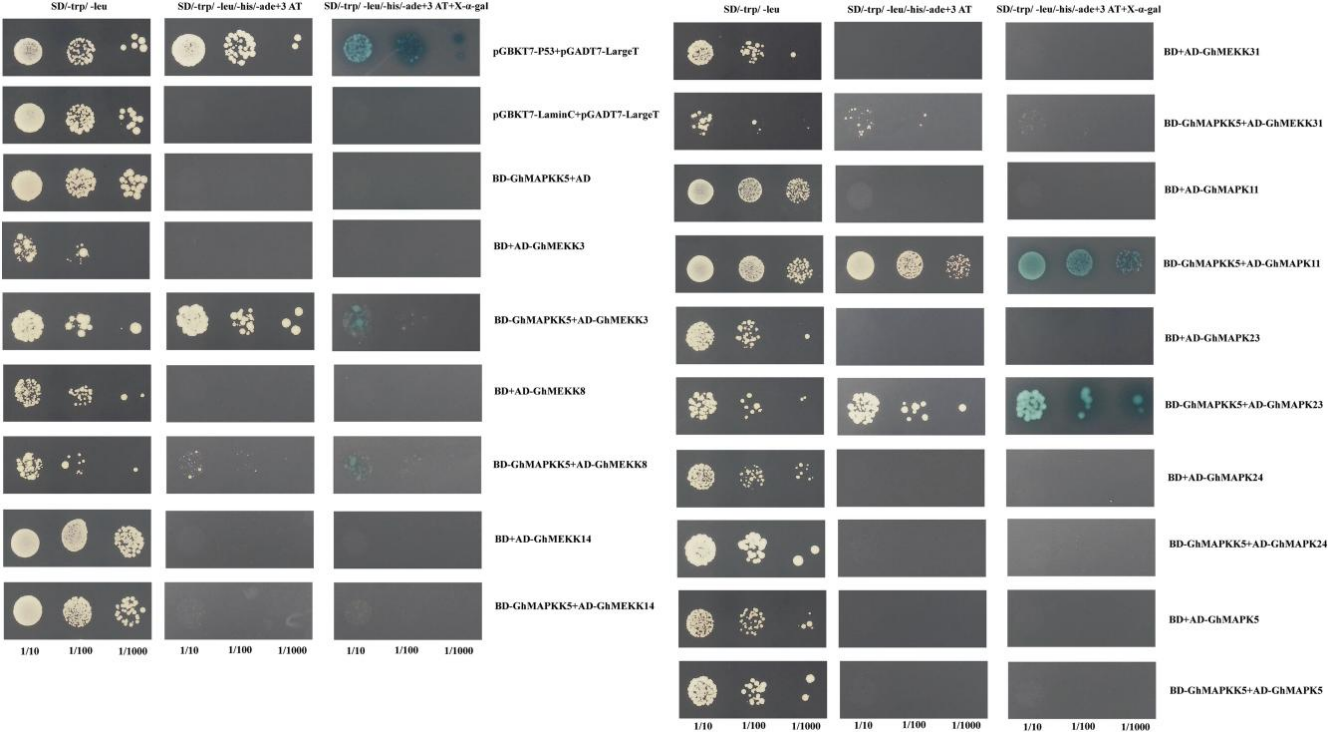
Y2H validation of the interaction between GhMAPKK5 protein and interacting proteins. Positive control: pGBKT7-p53 + pGADT7-LargeT; negative control: pGBKT7-LaminC + pGADT7-LargeT. The rest are experimental groups. AD (pGADT7); BD (pGBKT7).

We use the STRING network to predict the protein interaction network. *GhMAPKK5* is predicted to interact with *GhMAPK1* (*Gh_A07G1297*), *GhMAPK23* (*Gh_D08G1467*), and *GhMAPK24* (*Gh_A08G1183*) (Supplementary Fig. S10). To identify potential interaction partners by GhMAPKK5, we employed the Y2H system to probe interactions between GhMAPKK5 and eight candidate proteins: GhMEKK3/8/14/31 and GhMAPK5/11/23/24 (Fig. 9). Initial tests confirmed that GhMAPKK5 did not exhibit self-activation activity in the binding domain when inhibited by 3 AT. In exploring MAPKK–MAPK interactions, cells expressing *GhMAPKK5* with *GhMAPK11* and *GhMAPK23* grew on SD-4 medium and turned blue, suggesting possible interactions that could facilitate MAPK signaling. Additionally, interactions were observed in MEKK–MAPKK pairs notably between GhMEKK3/GhMAPKK5 and GhMEKK8/GhMAPKK5. In addition, GhMEKK31 has a weak interaction with GhMAPKK5. Drawing from the homology with Arabidopsis proteins and their interactions, it can be inferred that the function of the GhMEKK3/8/31–GhMAPKK5–GhMAPK11/23 cascade in cotton might parallel the AtMEKK1–AtMKK2–AtMPK4 cascade in Arabidopsis.

## Discussion

The MAPK signaling cascade serves as the most primitive signal transduction pathway in plants (Wen et al. 2023), where *MAPKKs* provide a key intermediate link between upstream and downstream of this cascade. Here, we analyzed *MAPKK* genes from 66 different species, including commercially cultivated cottons (Supplementary Table S7). Cotton harbors a notably higher count of *MAPKK* members compared to other plant species, with findings showing no direct link between the number of these genes and the genome size (Fig. 1). Cottons' MAPKKs possess a consensus sequence of the signature motif VGTxxYMSPER, the plant-specific phosphorylation target site motif, -S/TxxxxxS/T-, and the conserved aspartic acid and lysine residues within the active site motif (-D (L/I/V) K-) (Xu and Zhang 2015; Enders et al. 2017). A total of 488 MAPKK proteins were identified in our extensive analysis and categorized into five main phylogenetic divisions, a classification that aligns with their conserved motifs and structural characteristics. It is noteworthy that the MAPKK7/8/9/10 protein sequences were previously reported in Group D (Hamel et al. 2006), whereas our results suggested that the MAPKK10 model possesses an incomplete activation loop motif in which the 5′- S or T residue is either absent or located three to five residues upstream of the canonical position (Supplementary Fig. S1). It is, therefore, plausible to divide MAPKK7/8/9/10 into two subgroups, congruent with a previous study (Jiang and Chu 2018). Through the study of the domain in cotton, most of the genes in Group E belong to Pkc_like superfamily, and their functions may change (Supplementary Fig. S3). This high gene count is part of a broader trend where cotton's MAPKK families, much like those in *T. cacao* (Yapeng et al. 2021), trace back to a shared ancestral lineage, highlighting the evolutionary trajectory and gene duplication as key factors in the MAPKK gene family's expansion across species (Figs. 1 and 2). Bioinformatic analysis of *cis-*acting elements reveals the possible regulatory elements, which *MAPKK* genes from four cotton species may possess (Fig. 3). Further gene expression analysis shed light on the functional dynamics of *GhMAPKK* genes from *G. hirsutum*, revealing their organ-specific expression and regulatory roles in response to various abiotic stresses (Fig. 4), which are critical for plant growth and survival. Among these, *GhMAPKK5* stood out for its pivotal role in enhancing drought and salt tolerance.

*AtMAPKK2* is a homologous gene of *GhMAPKK5*, which has been extensively studied in the past. AtMAPKK1/2 is involved in Arabidopsis' defense response to jasmonate and salicylate dependence (Qiu et al. 2008). AtMAPKK2 affects the vein structure of plants by affecting auxin transport patterns (Stanko et al. 2014). Enhanced freezing and salt tolerance was observed in *AtMAPKK2* overexpression in Arabidopsis plants (Teige et al. 2004; Hua et al. 2006). The excessive expression of *PeMKK2a* in poplar increased the salt tolerance of poplar (Wang et al. 2022). Similar to *AtMAPKK2*, the VIGS technique was used to silence *GhMAPKK5* genes in cotton, and it was found that PYL156: *GhMAPKK5*-silenced cotton plants suffered more wilting than control plants when treated with 150 mM NaCl and 18% PEG (Fig. 5). Moreover, transgenic Arabidopsis plants overexpressing *GhMAPKK5* performed better under salt and drought conditions (Fig. 8).

Comparative transcriptomic data indicated large amounts of DEGs following drought and salt treatments. The GO analysis showed that those DEGs were enriched in processes related to organic nitrogen compound catabolism, amino acid biosynthesis, cellular metabolism, and cellular protein metabolism (Supplementary Fig. S6). Different terms associated with cell composition and molecular functions, such as plastid formation, protein kinase activity, and carbohydrate derivative binding, were identified in both VIGS and control groups, highlighting their involvement in these processes. Under drought and salt stress, the biological processes were mainly enriched in regulation of gene transcription, phosphorus metabolic process, protein phosphorylation, carboxylic acid metabolic process, and organic acid metabolic process. A significant number of genes encoding TFs showed differential expression in response to drought and salt stress. Among them, 50 *WRKYs*, 56 *MYBs*, 56 *AP2-EREBPs*, four *bZIPs*, and 45 *bHLHs* were differentially expressed, either due to *GhMAPKK5* silencing or stress treatment (Supplementary Table S5). Notably, the majority of *WRKYs* were downregulated, whereas most *bHLH*s were upregulated under stress conditions, indicating a differential expression pattern. These TFs include *MYBs* involved in cell differentiation, root development, drought, stress, and hormone signaling (Huang et al. 2008; Sajjad et al. 2021), *bZIPs* associated with plant immune responses, and *WRKYs* playing roles in hypoxia, dehydration, ethylene, jasmonic acid, and plant growth regulation (Sajjad et al. 2021). This suggested a complex network where *GhMAPKK5* coordinates with these factors to modulate drought and salinity tolerance in plants.

By leveraging KEGG pathway enrichment analysis, we found that DEGs from the comparison of CK *vs.* VIGS stood in the pathways related to plant–pathogen interaction, MAPK signaling pathway, plant hormone signal transduction, carbon fixation in photosynthetic organisms, amino acid degradation, glucose metabolism, and biosynthesis of unsaturated fatty acids (Fig. 6 and Supplementary Fig. S7). These pathways have been shown associated with abiotic stress tolerance (Han et al. 2023; Mishra et al. 2024; Zaman et al. 2023). These secondary metabolic networks interact to regulate the elimination of ROS to overcome drought and salt stress (Verma et al. 2020; Ali et al. 2021; Liu et al. 2021), supported by our transcriptomic data (Fig. 6). In addition, the silencing of *GhMAPKK5* impaired key genes in ABA signaling pathways-associated, RALF and proline synthesis, and transport pathways (Fig. 7). GhMEKK3/GhMEKK8/GhMEKK31/GhMAPK11/GhMAPK23 interacts with GhMAPKK5 as verified by Y2H (Fig. 9). In Arabidopsis, the MEKK1–MKK2–MPK4/MPK6 cascade was induced by salt and cold stress and impacted the downstream of ROS (MAPK Group 2002; Teige et al. 2004; Zhang et al. 2016). In this case, it is worthwhile to examine whether GhMEKK3/GhMEKK8/GhMEKK31–GhMAPKK5–GhMAPK11/23 may form complex to mirror AtMEKK1–AtMAKK1/2–MPK4/MPK6 from Arabidopsis in the future, which is expected to provide a broader perspective on the evolutionary conservation and functional importance of these signaling pathways in plant stress responses.

**Figure 10.**
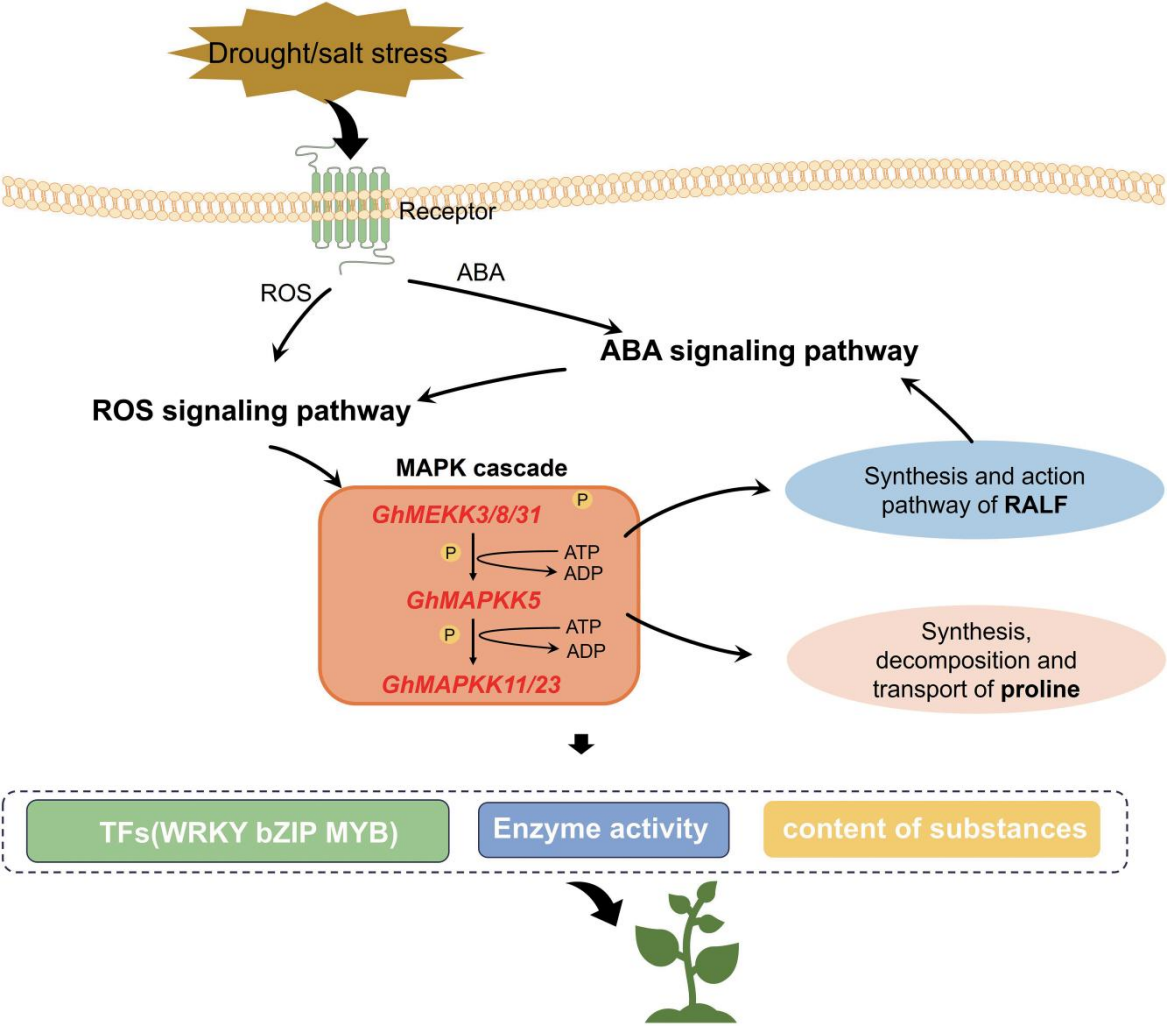
Regulatory network model of cotton plant response to drought stress, salt stress, and various exogenous substances. The contents of ROS and ABA in plants under drought stress and salt stress change and activate the ROS and ABA signaling pathway. Studies have shown that ROS products can activate the MAPK signaling pathway, and the silencing of *GhMAPKK5* gene in the MAPK signaling pathway will affect the expression of upstream and downstream genes. The cascade GhMEKK3/GhMEKK8/GhMEKK31–GhMAPKK5–GhMAPK11/23 associated with GhMAPKK5 was identified by Y-2H. This cascade leads to cell homeostasis by affecting upregulated ROS-clearing enzymes (SOD, CAT, POD) and genes associated with proline and RALF synthesis and transport pathways. ROS (reactive oxygen species); ABA (abscisic acid); MEKK (mitogen-activated protein kinase kinase kinase); MAPKK (mitogen-activated protein kinase kinase); MAPK (mitogen-activated protein kinase); RALF (rapid alkalinization factor).

The additional supplement of ABA, proline, and small peptides alleviated the sensitive response of *GhMAPKK5* silencing cottons to drought and salt treatments that had good correlation with the activity of ROS-clearing enzymes SOD, CAT, and POD (Fig. 7), suggesting *GhMAPKK5*-mediated stress response may involve the regulation of ROS production in cotton. ROS can be produced through a variety of cellular processes in plants with an important role in response of plant defense to stress, such as drought and salt stresses (Martinez et al. 2020; Liu et al. 2022). In previous studies, MAPK signaling can interact with ROS and ABA signaling for mitigating the impact of abiotic stress (Manna et al. 2023). Enhancing ROS production can activate MAPK signaling in plants, such as *AtMAPK1*, *AtMAPK2*, *AtMAPK3/4/6/7*, *AtMEKK1* (a *MAPKK*), or *ANP1* (a *MAPKKK*) when exogenous hydrogen peroxide (H_2_O_2_) was applied to Arabidopsis (González 2023). In addition to H_2_O_2_, ozone treatment and a shift in cellular redox potential also led to activation of the MAPK cascade in plants (Ali et al. 2021). The indirect activation of ROS-scavenging MAPK signals initiates a feedback mechanism that upregulates related ROS-clearing enzymes (SOD, CAT, POD) by influencing the expression of downstream genes, leading to cell homeostasis (Waadt et al. 2022; Majeed et al. 2023). ABA is a key hormone involved in plant growth, development, and stress adaptation via either ABA receptor-mediated post translational regulation or ABA-triggered transcriptional regulation (Ali et al. 2021). In previous studies, *AtMAPK9/12* functioned downstream of ROS to positively regulate cellular ABA signaling (Fabien Jammesa et al. 2009). Plant ABA receptors known as *PYR/PYL/RCAR* were significantly downregulated after silencing the *GhMAPKK5* in cotton. When plants experience osmotic stress, the contents of osmoregulatory substances in cells help in improving tolerance to stress (Ozturk et al. 2021 ). Among these, the accumulation of proline cannot only regulate the pressure inside and outside the cell as an osmotic regulator, but also has the ability of active oxygen removal, which can improve the plant stress resistance in many ways (Verma et al. 2020). The change of proline content was mainly affected by *P5CS*, *P5CR*, *ProDH* and *ProT* (Yang et al. 2021 ). In transcriptome data, there were significant changes in related genes *PC5S* (*PYCS*), *PC5R* (*PYCR*), *ProDH*, and *ProT* from cotton, which were consistent with RT-qPCR analysis. In our previous research, we have found that *GhRALFs* are a negative regulator of salt tolerance in cotton (Lin et al. 2022). Interestingly, our experiments in this research indicated that exogenous supplement of RALF alleviated the stress-sensitive performance caused by *GhMAPKK5* silencing (Fig. 7), suggesting that *GhMAPKK5* may intermediate *GhRALFs*' function, and the lack of *GhMAPKK5* may deepen the negative effects of *GhRALFs*. Consistently, silencing *GhMAPKK5* reduced the expression of RALF receptor gene *FER*, which in turn can regulate the activity of protein kinase PP2C through ABA-insensitive (ABI) protein, thereby may affecting ABA signaling pathways. Moreover, the addition of exogenous substances will affect the seed germination, survival, and root development of transgenic Arabidopsis. The overexpression of *GhMAPKK5* in Arabidopsis can affect the expression of related genes, such as *AtFER*, *AtSnRK*, and *AtPYL*. The summary, as shown in Fig. 10, hints at a sophisticated model where the *GhMAPKK5* orchestrates a network involving ABA, ROS, and RALF peptides to enhance cotton plant resilience to drought and salt stress. This intricate network points towards a holistic strategy employed by plants to manage the detrimental effects of abiotic stresses.

## Conclusions

This research analyzed the genome-wide identification of *MAPKKs* in 66 different species. The conserved motif analysis, chromosome localization, collinearity analysis, *Ka*/*Ks* analysis, gene structure analysis, and promoter *cis*-acting element analysis were investigated in cotton. The regulation of *MAPKK* genes from Arabidopsis, rice, and cotton responses to abiotic stress was examined. Further studies in plants have shown that *GhMAPKK5* is a positive regulator of cotton stress resistance possibly through coordination of the ABA, ROS, and RALF peptide networks through the GhMEKK3/GhMEKK8/GhMEKK31–GhMAPKK5–GhMAPK11/23 cascade. This finding opens avenues for research into MAPKK protein family and provides a valuable genetic target for engineering stress-resistant crop varieties.

## Materials and methods

### Sequence acquisition and identification of MAPKK family genes

Genomic files of *G. hirsutum* (*Gossypium hirsutum*) (NAU), *G. arboretum* (*Gossypium arboretum*) (CRI), *G. raimondii* (*Gossypium raimondii*) (JGI), and *G. barbadense* (*Gossypium barbadense*) (HAU) were downloaded from CottonFGD (https://cottonfgd.net/). The MAPKK proteins from Arabidopsis (*Arabidopsis thaliana*) and rice (*Oryza sativa*) were retrieved from TAIR (https://www.arabidopsis.org/) and the Rice Genome Annotation Project (http://rice.plantbiology.msu.edu/). The genomic and protein sequences of other species were obtained from the public database NCBI. The full-length sequence of AtMAPKK proteins was used as a query. Then, a BLAST search was carried out using BLASTP at a threshold value (e-value ≤1 × 10^−5^), and the aligned parts were inspected and compared manually to determine their identity. The candidate gene sequences were further identified by verifying the conservative protein kinase domain (PS50011), PK ATP-binding signature (PS00107), and S/T PK active-site signature (PS00108) of the sequences on ExPASy PROSITE (https://prosite.expasy.org/). The MAPKK sequences showing unknown conserved domains were rejected.

### Bioinformatic analysis

The physical and chemical properties of the MAPKK proteins, including the number of amino acids, isoelectric point (*pI*), molecular weight (*M_W_*), subcellular localization, and hydrophilicity, were predicted by using the ExPASy server (https://web.expasy.org/compute_pi/), SignalP-5.0 (https://services.healthtech.dtu.dk/services/SignalP-5.0/), and ProtScale (https://web.expasy.org/protscale/).

Multiple sequence alignments of the identified protein sequences were carried out by deploying Clustal W2. The phylogenetic trees were constructed from the MAPKK proteins through employing NJ method (with 1,000 replications) using MEGA7. Substitution method chose *p*-distance, and missing data were processed using pairwise deletions. Other parameters were kept as default. Phylogenetic trees were grouped according to published literature (MAPK Group 2002), and motif analyses were performed on the sequences of each grouping using Multiple Em for Motif Elicitation (MEME, https://meme-suite.org/meme/tools/meme).

Interspecific collinearity analysis was performed on Arabidopsis, *G. hirsutum*, *O. sativa*, and *G. arboreum*, *G. hirsutum*, and *G. raimondii*. The MCScanX tool was used to analyze the whole genome sequences and annotation files of *G. hirsutum*, *O. sativa*, and four cotton species. Their collinear and homologous chromosomal regions were visualized, and the repeated genes of four cotton species were highlighted by TBtools. To investigate the selection pressure experienced by *MAPKK* duplicated gene pairs belonging to Arabidopsis, *G. hirsutum*, *O. sativa*, and a few lower plants, including *bryophytes*, *pteridophyte*, and *basal angiosperm*, the rates of synonymous (*Ks*) and non-synonymous (*Ka*) substitutions along with their ratios were calculated by the *Ka*/*Ks* calculator in TBtools.

The coding sequencing (CDS) and genomic sequences of four cotton species were used to draw the diagram of exon/intron distribution with the NWK file of the phylogenetic tree at the Gene Structure Display Server (GSDS, http://gsds.cbi.pku.edu.cn/). Chromosome locations of these genes on *G. arboreum*, *G. hirsutum*, *G. raimondii*, and *G. barbadense* were determined using TBtools. The analysis of the conserved domain of gene family was performed by the Conserved Domain Database (CDD) of NCBI (https://www.ncbi.nlm.nih.gov/Structure/cdd/cdd.shtml). After retrieving conserve domain, TBtools were used to visualize NCBI CDD domain pattern function.

To study the regulatory elements of *MAPKKs*, the upstream genomic sequences (2.0 kb) of each gene were extracted by TBtools. These sequences were searched against the PlantCARE database (http://bioinformatics.psb.ugent.be/webtools/plantcare/html/) to identify the *cis*-acting regulatory elements.

Gene ontology (GO) enrichment analysis and the Kyoto Encyclopedia of Genes and Genomes (KEGG) pathway analysis were performed to gain insights into the biological processes, molecular functions, and pathways associated with differentially expressed genes. GO analysis was performed in R software using the gene ontology resource database. For KEGG pathway annotation, phyper function in R software was used for enrichment analysis; *P*-values were computed; and then a false discovery rate (FDR) correction was performed on these *P*-values. Functional categories exhibiting q-value ≤0.05 were considered to have significant enrichment.

### Plant materials, growth conditions, and abiotic stress treatment

For conducting these experiments, a drought-tolerant and salt-tolerant cotton variety (*G. hirsutum* L. cv. Tahe-2) was selected. Cotton tissues used in the experiment, such as petal, stamen, pistil, calycle, ovules, and fiber, were sampled from the East Yard at the Cotton Institute of the Chinese Academy of Agricultural Sciences, Anyang, Henan, China. After soaking in ddH_2_O for 3 to 5 h, the cotton seeds were sown in sandy soil and grown to the three-leaf stage under greenhouse conditions (14 h light (28 to 30 °C)/10 h dark (25 to 28 °C), 150 µmol m^−2^ s^−1^).

The seedlings of *Japonica rice* (*O. sativa japonica*) were sown on sterilized substrate (vermiculite: nutrient soil = 1:1) at 28 °C for 72 h prior to shifting in an incubator under 16 h light and 8 h dark at 28 °C for 2 weeks. Seeds of *Arabidopsis ecotype Columbia 0* were sterilized with 75% (v/v) alcohol, then washed with sterilized ddH_2_O and cultures on 1/2 Murashige and Skoog (MS) medium. Two days after vernalization at 4 ℃, seeds were placed in an incubator for 1 week under 16/8 h of light/dark cycle and 80% RH. Then, the seedlings with intact cotyledon leaves were transplanted into pots containing sterilized substrate (vermiculite: nutrient soil = 1:1) and placed in glasshouses for 1 weeks for experimental treatment.

Similar growth features of cotton, rice, and Arabidopsis were subjected to different treatment. The control group was exposed to ddH_2_O, 25 °C (control) for 24 h; the drought (osmotic) treatment group was treated with 18% PEG 6000 solution at 25 °C for 24 h; salt stress treatment group was treated with 150 mM NaCl solution at 25 °C for 24 h; the high-temperature treatment group (cotton) was exposed to 40 °C for 24 h; and the low-temperature treatment group (cotton) was exposed to 4 °C for 24 h. Three biological replicates were performed for each experiment with 15 plants per replicate. At the treatment time of 1, 3, 6, 12, and 24 h, the leaves of each group of seedlings were collected, rapidly frozen in liquid nitrogen, and stored at −80 ℃ for RNA extraction.

### Expression analyses of the *MAPKK* genes

The expression patterns of *MAPKKs* in Arabidopsis, *G. hirsutum*, and *O. sativa* were studied in different tissues and under various abiotic stresses. The expression profiles of *AtMAPKKs* and *OsMAPKKs* during different developmental stages and tissues were obtained from the Arabidopsis database TAIR and Rice eFP browsers in the Bio-Analytic Resource database (http://bar.utoronto.ca/). In *G. hirsutum*, the expression of *MAPKKs* in different tissues as well as under abiotic stresses (PEG, salt, high and low temperatures) was obtained from transcriptome data. RNA seq data were obtained from the NCBI Sequence Read Archive (SRA: PRJNA248163) (Zhang et al. 2015).

Total RNA was extracted using the EASYspin Plus Plant RNA Kit (Aidlab, Peking, China) by adopting the procedure described by the manufacturer. Complementary first-strand DNA (cDNA) was obtained using the Prime Script RT Reagent Kit (Perfect Real Time, Takara, Dalian, China). The cDNA samples were diluted five-fold with ddH_2_O for qPCR (quantitative real-time PCR) on the ABI ™6 Flex platform. Primers were designed using online tools in GenScript website (https://www.genscript.com/tools/real-time-pcr-taqman-primer-design-tool) according to primer design principles, and the specificity of primers was detected by Primer-BLAST in NCBI (https://www.ncbi.nlm.nih.gov/tools/primer-blast/index.cgi) (Supplementary Table S8). *GhActin* (*AY305733*), *OsActin* (*AK100267*), and *AtActin2* (*AT3G18780*) were used as reference genes, and the 2^−△△CT^ method was used to calculate the relative expression of *MAPKK* genes for each sample. *MAPKK* expression was analyzed in 2.3 samples under stress. Twelve upland cotton (*G. hirsutum* L. cv.) accessions, namely, TM-1, KF868, Tahe-2, XQ-1, Han5158, CCRI9001, YS6, LMY16, Han117, LM6, CCRI12, and CCRI49, were used to determine the expression of *GhMAPKK5* during drought/salt treatment.

### VIGS experiment of *GhMAKK5*

Full-length cDNA of *GhMAKK5* (*Gh_A07G0124.1*) was amplified using primers (Supplementary Table S8) containing *Bam*HI and *Sac*I restriction sites on their respective 5′-ends. The purified *GhMAPKK5* fragments were cloned into pYL156. The GV3101 strains carrying pYL156 (empty vector), pYL156: *GhMAPKK5* (VIGS), pYL156: PDS (positive control), and pYL192 (helper vector) were cultured to achieve the 1.2 to 1.5 OD_600_ values. The bacterial cells were collected by centrifugation, and then resuspended in an appropriate volume of the resuspension to a final concentration of OD_600_ 1.0. The inoculum containing pYL192 and *A. tumefaciens* harboring pYL156 (1:1 ratio) was incubated at 23 °C for 3 to 4 h in the dark. After the incubation, cotyledons of cotton were infected with the mixed bacterial solution. Afterwards, the seedlings were placed in the dark overnight, followed by providing 16-h light/8-h dark cycle at 25 °C. The VIGS experiment was successful when plants injected with pYL156: PDS showed albino phenotype, and the silencing of *GhMAPKK5* gene was confirmed by RT-qPCR. At the three-leaf stage, drought and salt treatment were carried out, with the same treatment conditions as 2.3, and sampling was carried out after 6 h of treatment. Malondialdehyde content and catalase activity were determined with the kit (MDA: Cat: BC0025-100T/96S; CAT: Cat: BC0205-100T/96S), and the leaf samples were sent to BGI Co. (Shenzhen, China) for transcriptome sequencing.

### Application of different exogenous substances (ABA, proline, and RALF) and examination of physiological indexes

In order to better understand the regulatory mechanism of *GhMAPKK5*, VIGS cotton plants were treated with ABA, proline, and small peptides. The synthesized small peptide RALF (Qiang Yao Biotechnology Co., Ltd.) was dissolved in 10% acetonitrile (ACN) and prepared into 100 μM solution, and then diluted to 100 μM with ddH_2_O. For the ABA, proline, and rapid alkalinization factor (RALF) response assay, 100 mM ABA, 100 μM proline, and 10 μM RALF solutions were evenly sprayed on the cotton leaves, and ddH_2_O was used as a control. Three biological replicates were performed for each experiment, with 15 cotton plants per replicate. The leaves were sampled at 6 h and harvested to analyze serial physiological parameters, including malondialdehyde (MDA), proline, catalase (CAT), peroxidase (POD), and superoxide dismutase (SOD), using the specific commercial kits (Beijing Solarbio Science & Technology Co., Ltd., MDA: Cat: BC0025-100T/96S; Pro: Cat: BC0295-100T/96S; CAT: Cat: BC0205-100T/96S; POD: Cat: BC0095-100T/96S; SOD: Cat: BC0175-100T/48S).

### Transformation of Arabidopsis, seed germination rate, survival rate, and root length assays

The ORFs (1089 bp) of *GhMAPKK5* were cloned under the control of the 35S promoter in the plant expression vector *pBI121*. The developed constructs were transformed into *Agrobacterium tumefaciens* (*strain GV3101*) and transferred into wild Arabidopsis Col-0 using the floral-dip method by soaking the inflorescence for 1 min (Clough and Bent 1998). The T_1_ seeds were screened in 1/2 MS culture medium containing 50 mg/L kanamycin.

Transgenic *GhMAPKK5* Arabidopsis seeds of T_3_ generations and WT were disinfected and uniformly seeded in 1/2 MS medium and 1/2 MS medium supplemented with 250 mM mannitol (Man) and 150 mM NaCl. The medium was vernalized at 4 °C for 2 days, and then transferred to an incubator for culture. The germination rate was calculated daily. The survival rate was calculated, and the phenotype was recorded after 7 days. The drought and salt tolerance of *GhMAPKK5* was investigated in T_3_ transgenic Arabidopsis and wild type at seedling stage under drought and salt treatments. The simulated drought was done without water for 15 days. Salt stress simulation processing with the method of salt solution to water gradually increased the concentration of salt solution (50, 100, and 150 mM) with 15 days. Phenotypes were observed and recorded. In addition, exogenous substances 10 μM proline, 5 μM RALF, and 0.5 μM ABA were added to 150 mM NaCl and 250 mM mannitol 1/2 MS medium, and the germination rate, survival rate, and root length of transgenic Arabidopsis and WT seeds were calculated.

### Yeast two-hybrid (Y2H) assays

Y2H was determined using the Matchmaker Gold Yeast Two-Hybrid System (Clontech). The full-length CDS of *GhMEKK3*, *GhMEKK8*, *GhMEKK14*, *GhMEKK31*, *GhMAPK5*, *GhMAPK11*, *GhMAPK23* and *GhMAPK24* were constructed into yeast vector pGADT7. The full-length CDS sequence of *GhMAPKK5* was cloned into vector pGBKT7 and introduced into yeast strain *AH109*. Subsequently, the self-activation activity of the decoy protein pGBKT7-GhMAPKK5 was detected, and its self-activation was inhibited with 2.5 μM 3AT (3-amino-1,2,4-triazole, a commonly used competitive inhibitor of yeast dihybrid, inhibits self-activation). Interactions between different proteins were identified as SD-trp-leu (SD-2), and SD-trp, -leu, -his, -ade (SD-4) (with X-a-Gal) grown on SD medium. Co-transformation of pGBKT7-p53/pGADT7-largeT and pGBKT7-laminC/pGADT7-largeT served as positive and negative controls, respectively.

### Statistical analysis of data

Each experiment had three biological replicates, and each biological replicate contained three technical replications. GraphPad Prism 9.0 was used for plotting the data, and the significance test of the data was carried out with SPSS software (version 26.0). The value bars with ns are not significant (ns omit), and the asterisk denotes statistically significant differences, *P < 0.05, **P < 0.01, and ***P < 0.001.

### Accession numbers

Sequence data from this article can be found in the GenBank/EMBL data libraries under accession numbers listed in Supplementary Table S7, where the *MAPKK* gene in cotton is found in the cottonFGD database.

## Supplementary Material

kiae415_Supplementary_Data

## Data Availability

The data underlying this article will be shared on reasonable request to the corresponding author.

## Supplementary data

The following materials are available in the online version of this article.

**Supplementary Figure S1.** WebLogo3 shows the conserved regions of the five MAPKK subgroups.

**Supplementary Figure S2.** Chromosome mapping of *MAPKK* genes in four cotton species.

**Supplementary Figure S3.** Gene structure and motif analysis of *MAPKK* in four cotton seeds.

**Supplementary Figure S4.** Expression patterns of *MAPKKs* under different stress.

**Supplementary Figure S5.** Correlation heat map between samples. CK: pYL156; CK_D: pYL156 Drought 6 h; CK_S: pYL156 Salt 6 h; VIGS: pYL156: *GhMAPKK5*; VIGS_D: pYL156: *GhMAPKK5* Drought 6 h; and VIGS_S: pYL156: *GhMAPKK5* Salt 6 h. The color scale bars represent the direct correlation between different samples.

**Supplementary Figure S6.** Gene ontology (GO) enrichment analysis.

**Supplementary Figure S7.** KEGG metabolic pathway enrichment analysis.

**Supplementary Figure S8.** Effects of exogenous substances on transgenic *Arabidopsis thaliana* under drought conditions.

**Supplementary Figure S9.** Effects of exogenous substances on transgenic *Arabidopsis thaliana* under salt conditions.

**Supplementary Figure S10.** GhMAPKK5 protein interaction prediction map.

**Supplementary Table S1.** Summary of *MAPKK* genes in 65 species.

**Supplementary Table S2.** Molecular characterization of *MAPKK* genes in four cotton species.

**Supplementary Table S3.** Read quality statistics.

**Supplementary Table S4.** All diff_exp genes.

**Supplementary Table S5.** Differentially expressed response genes.

**Supplementary Table S6.** Differentially expressed response genes in ABA signaling pathway, MAPK signaling pathway, and the synthesis and transportation of proline.

**Supplementary Table S7.** Gene number in the phylogenetic tree and NCBI geneID corresponding table.

**Supplementary Table S8.** Sequence of primers.
