# Supplementary material for: Molecular traits of MAPK kinases and the regulatory mechanism of GhMAPKK5 alleviating drought/salt stress in cotton
Source: Plant Physiol. 2024 Aug 14;196(3):2030–47. doi: 10.1093/plphys/kiae415 (PMC11531841; doi:10.1093/plphys/kiae415)
Supplement: kiae415_Supplementary_Data [file kiae415_supplementary_data.zip › suppfigures.docx]

**
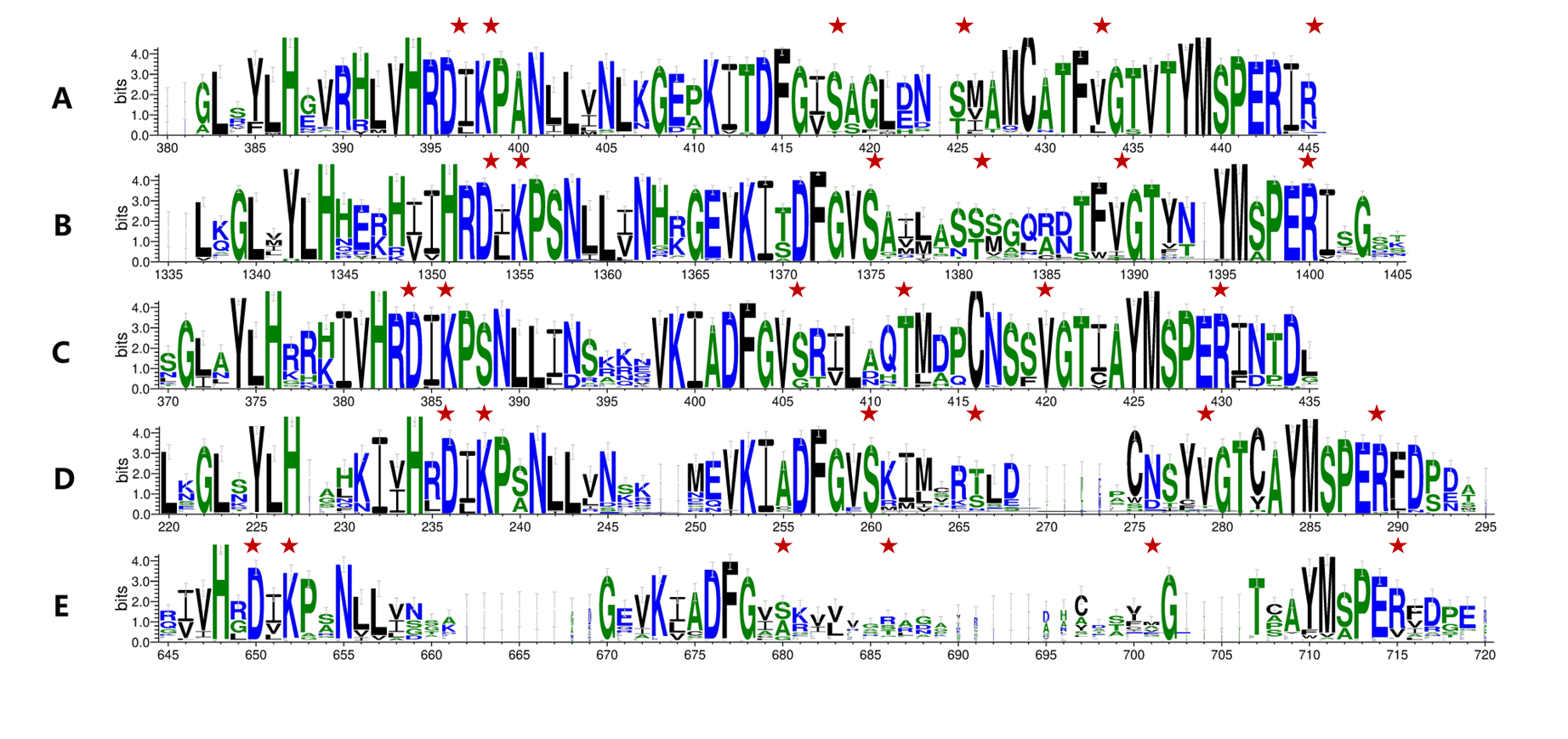
**

**Supplementary Figure S1** WebLogo3 shows the conserved regions of the five subgroups MAPKK. The regions between red stars indicate the presence of conserved aspartate and lysine residues D(L/I/V) K; Specific T/SXXXXXS structure and active sites such as -VG(E/T) XXYMSPER-. The horizontal axis is in bp.

**Supplementary Figure S2** Chromosome mapping of MAPKK genes in four cotton species. (A) *G. hirsutum*; (B) *G. barbadense*; (C) *G. arboretum*; (D) *G. raimondii*.


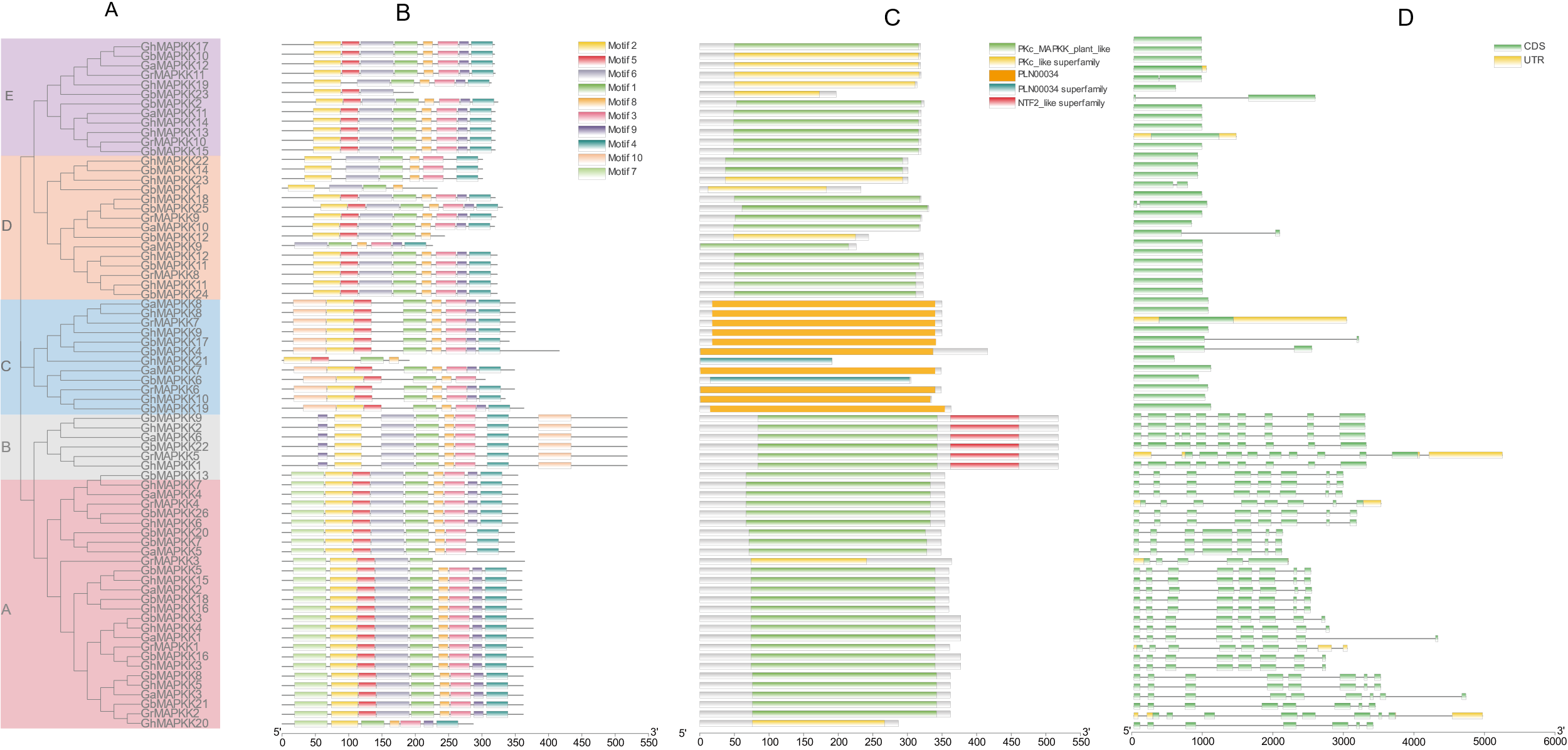


**Supplementary Figure S3** Gene structure and motif analysis of *MAPKK* in four cotton seeds. (A) Phylogenetic trees of MAPKK gene in four cottons were constructed using NJ method; (B) The motifs of MAPKK protein sequences of four cottons were analyzed by MEME; (C) The CDD of MAPKK protein sequences of four cottons were analyzed by NCBI; (D) Gene structure map of MAPKK gene in four cottons. CDS (coding sequences) are DNA sequences that correspond to the protein sequences one by one, and there are no other non-protein related sequences in the sequences. UTR (untranslated regions) is a non-coding region located on the 5' and 3' ends of mature ‌mRNA.


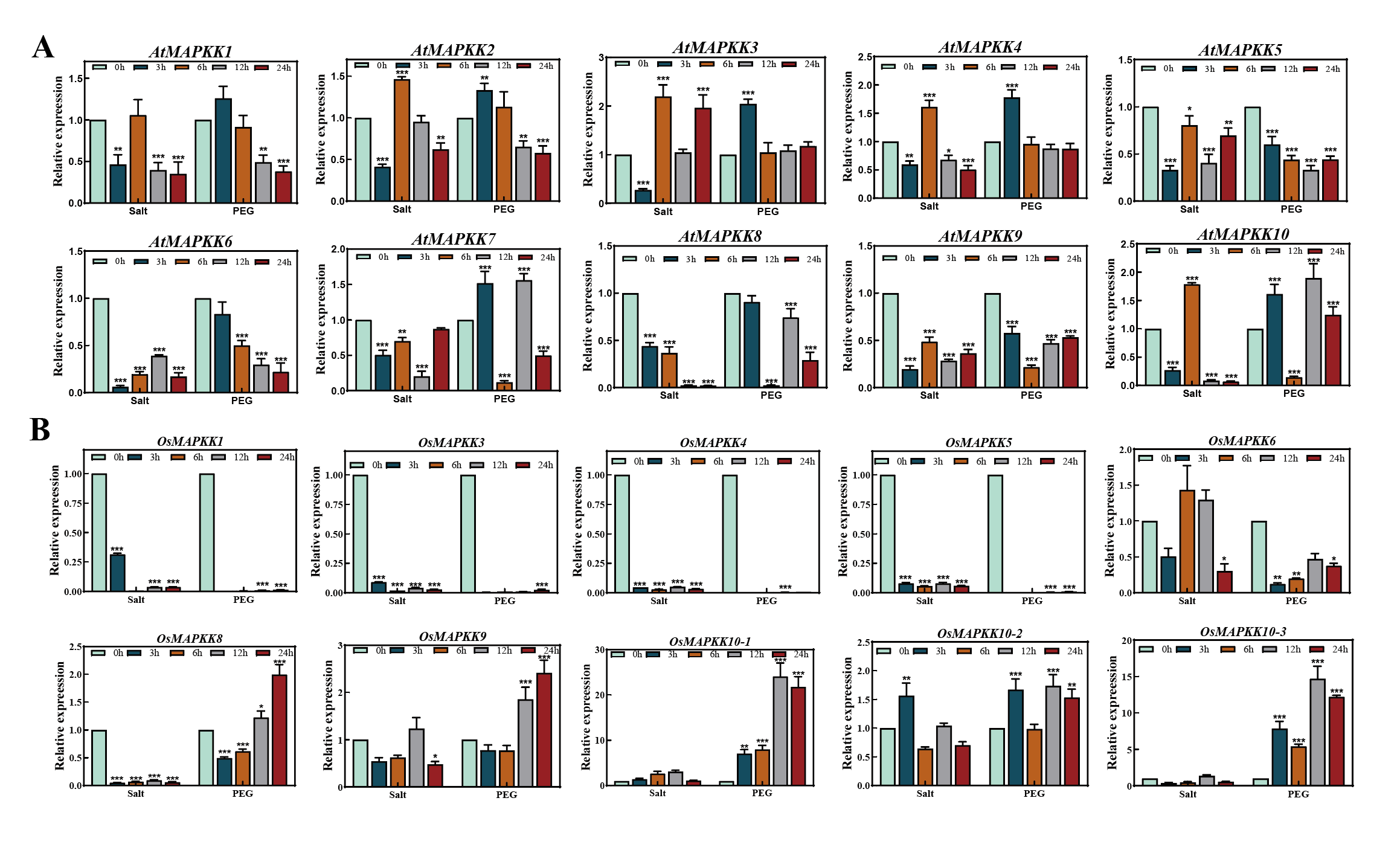


**Supplementary Figure S4** Expression patterns of MAPKKs under different stress. (A) Relative expression levels of 10 *AtMAPKK* genes under different stress. (B) Relative expression levels of 10 *OsMAPKK* genes under different stress. One-way analysis of variance was used. The error bars represent mean ± SD of at least three biological replicates (*p < 0.05, **p < 0.01, ***p < 0.001).


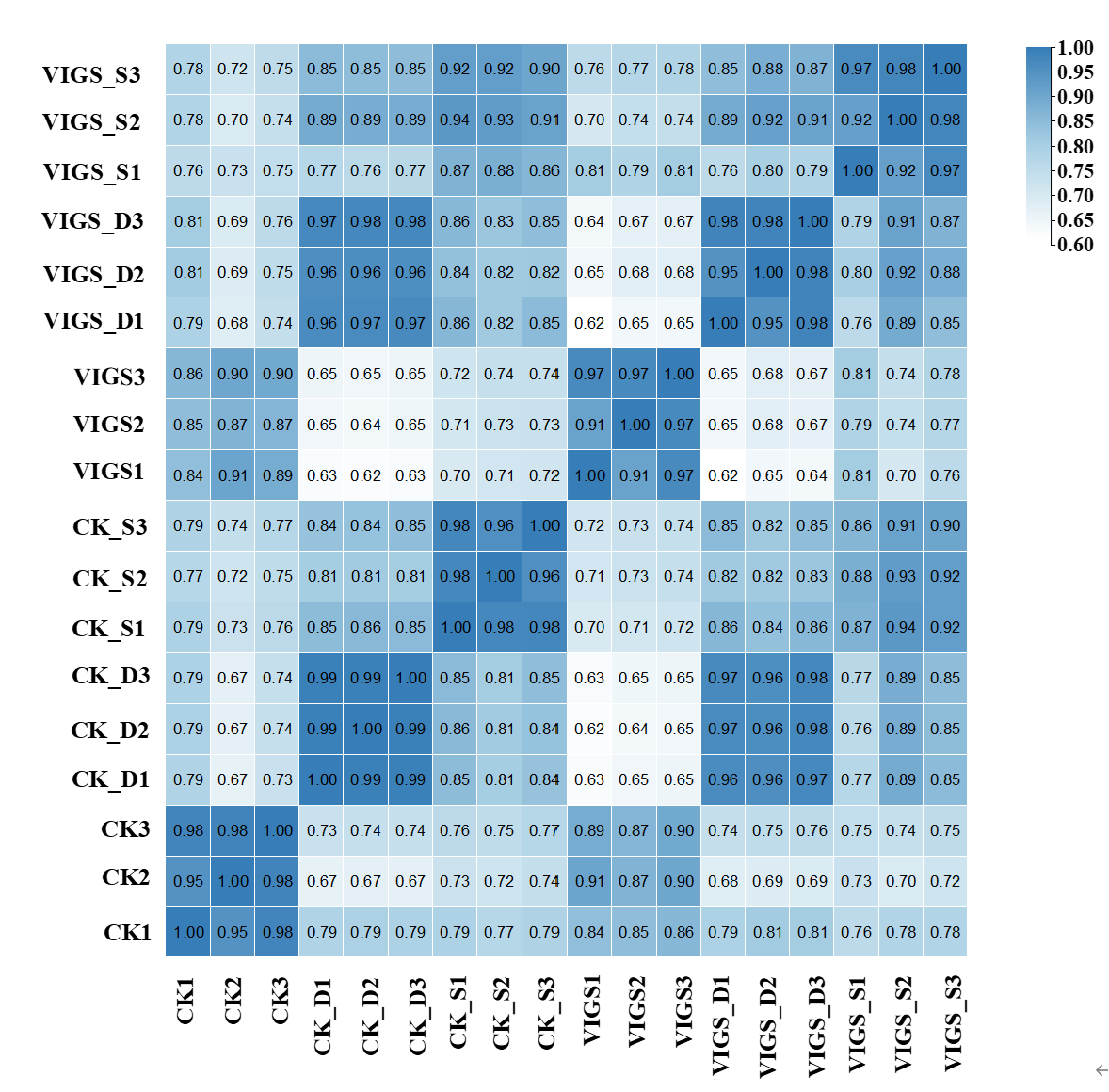


**Supplementary Figure S5** Correlation heat map between samples. CK: pYL156; CK_D: pYL156 Drought 6h; CK_S: pYL156 Salt 6h; VIGS: pYL156: GhMAPKK5; VIGS_D: pYL156: GhMAPKK5 Drought 6h; VIGS_S: pYL156: GhMAPKK5 Salt 6h. The color scale bars represent the direct correlation between different samples.


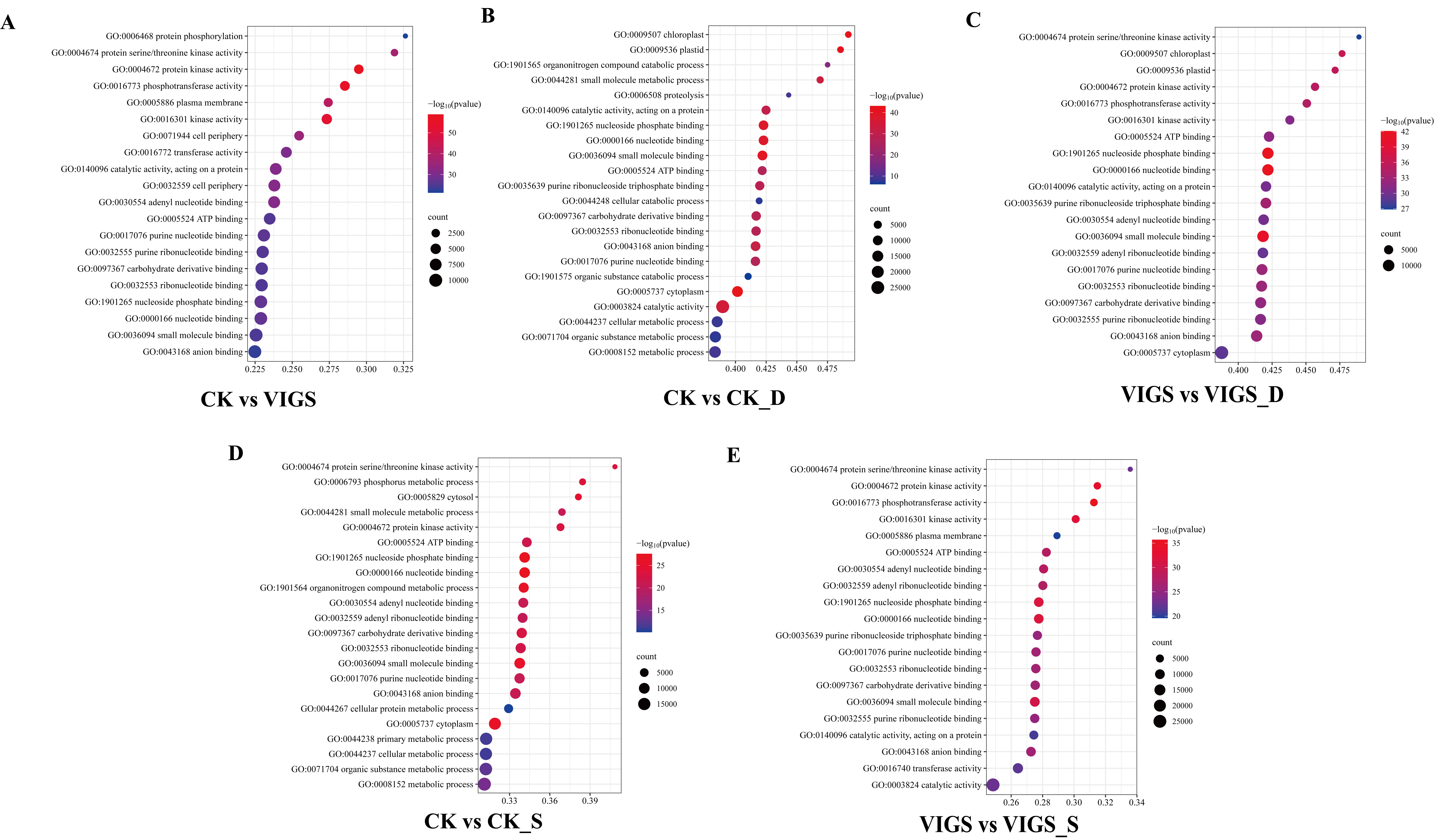


**Supplementary Figure S6** Gene ontology (GO) enrichment analysis. (A) GO terms of CK vs VIGS, (B) GO terms of CK vs CK_D, (C) GO terms of VIGS vs VIGS_D, (D) GO terms of CK vs CK_S, (E) GO terms of VIGS vs VIGS_S. Each circle in the figure represents a biological process, and the number of genes involved in a biological pathway corresponds to the size of the circle. The degree of significance of the GO-enrichment of DEGs is represented by q-value. The abscissa indicates the ratio of the number of DEGs annotated to a particular GO-term to the number of the DEGs annotated to all GO-terms.

CK= pYL156; CK_D= pYL156 drought 6h; CK_S= pYL156 salt 6h; VIGS= pYL156= GhMAPKK5; VIGS_D= pYL156= GhMAPKK5 drought 6h; VIGS_S= pYL156: GhMAPKK5 salt 6h


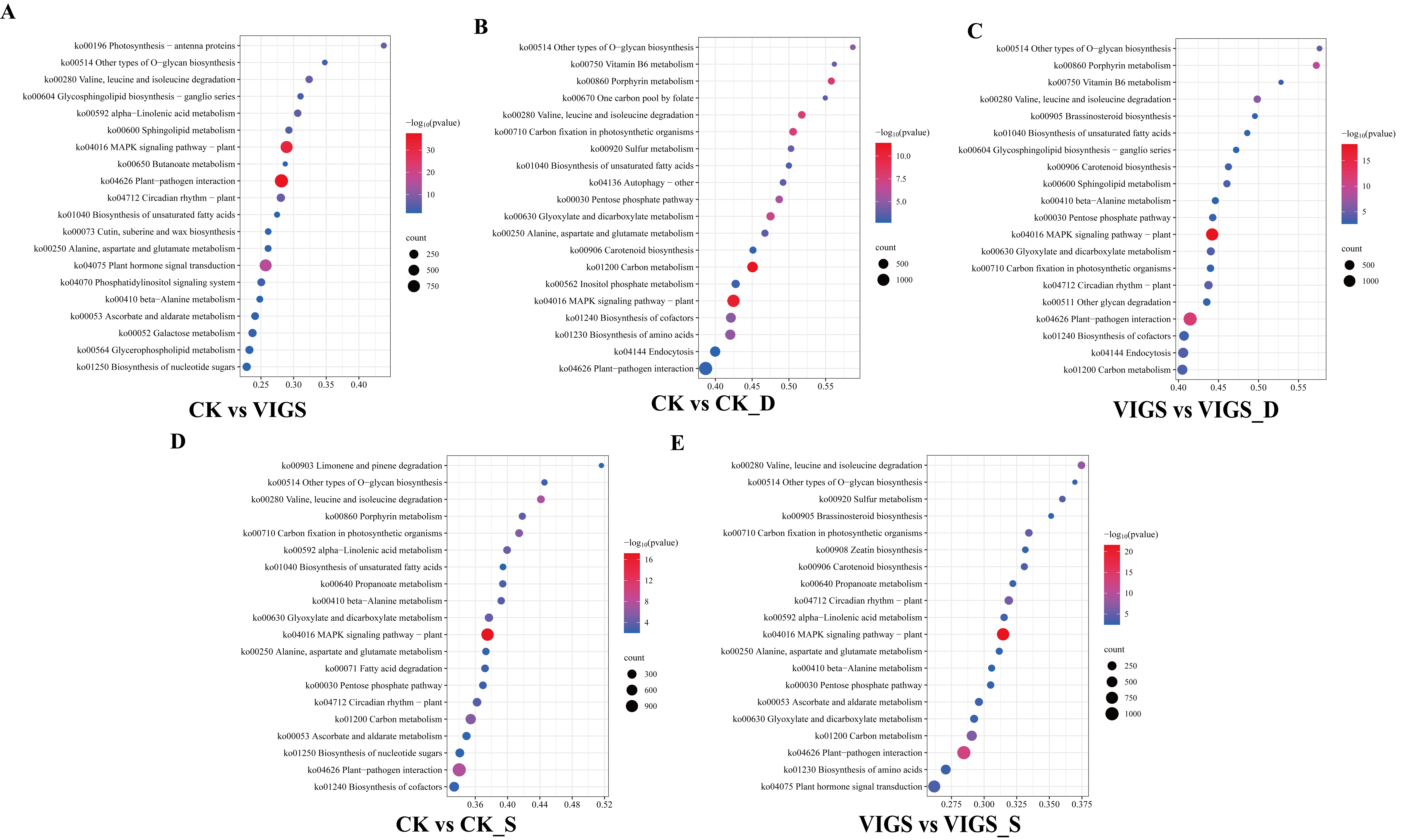


**Supplementary Figure S7** KEGG metabolic pathway enrichment analysis. (A) KEGG terms of CK vs VIGS; (B) KEGG terms of CK vs CK_D; (C) KEGG terms of VIGS vs VIGS_D; (D) KEGG terms of CK vs CK_S; (E) KEGG terms of VIGS vs VIGS_S. Each circle in the figure represents a KEGG biological process, and the number of genes enriched corresponds to the size of the circle. The degree of significance of the enrichment of DEGs is represented by qvalue. The abscissa indicates the ratio of the number of DEGs annotated to a particular KEGG term to the number of the DEGs annotated to all KEGG terms.

CK= pYL156; CK_D= pYL156 drought 6h; CK_S= pYL156 salt 6h; VIGS= pYL156: GhMAPKK5; VIGS_D= pYL156= GhMAPKK5 drought 6h; VIGS_S= pYL156: GhMAPKK5 salt 6h.


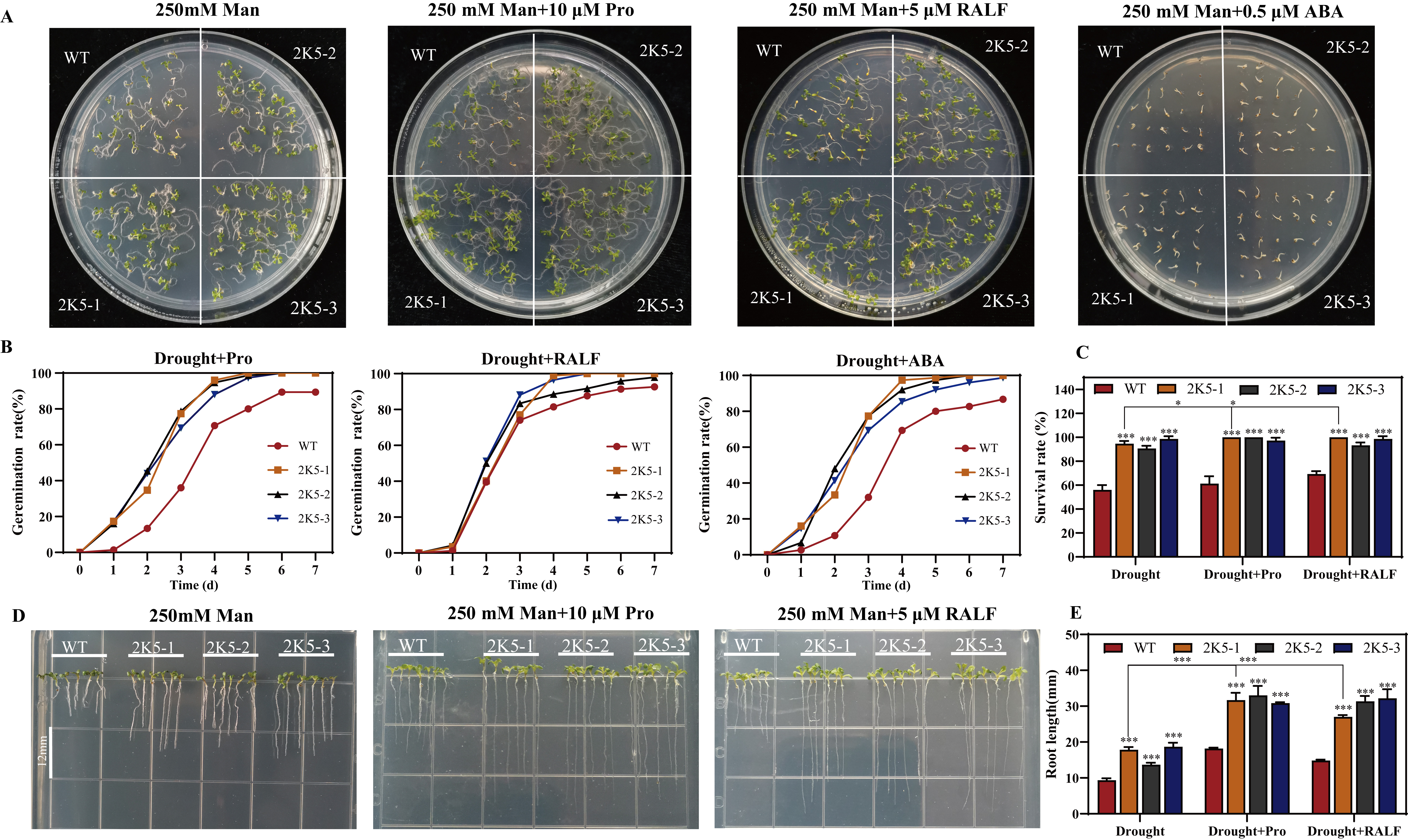


**Supplementary Figure S8** Effects of exogenous substances on transgenic *Arabidopsis thaliana* under drought conditions. (A, B) Germination of seeds under different conditions (C) Survival rate of seeds under different conditions; (D, E) Seedling root growth under different conditions. One-way analysis of variance was used. The error bars represent ± SD of at least three biological replicates (* p < 0.05, ** p< 0.01, *** p < 0.001).


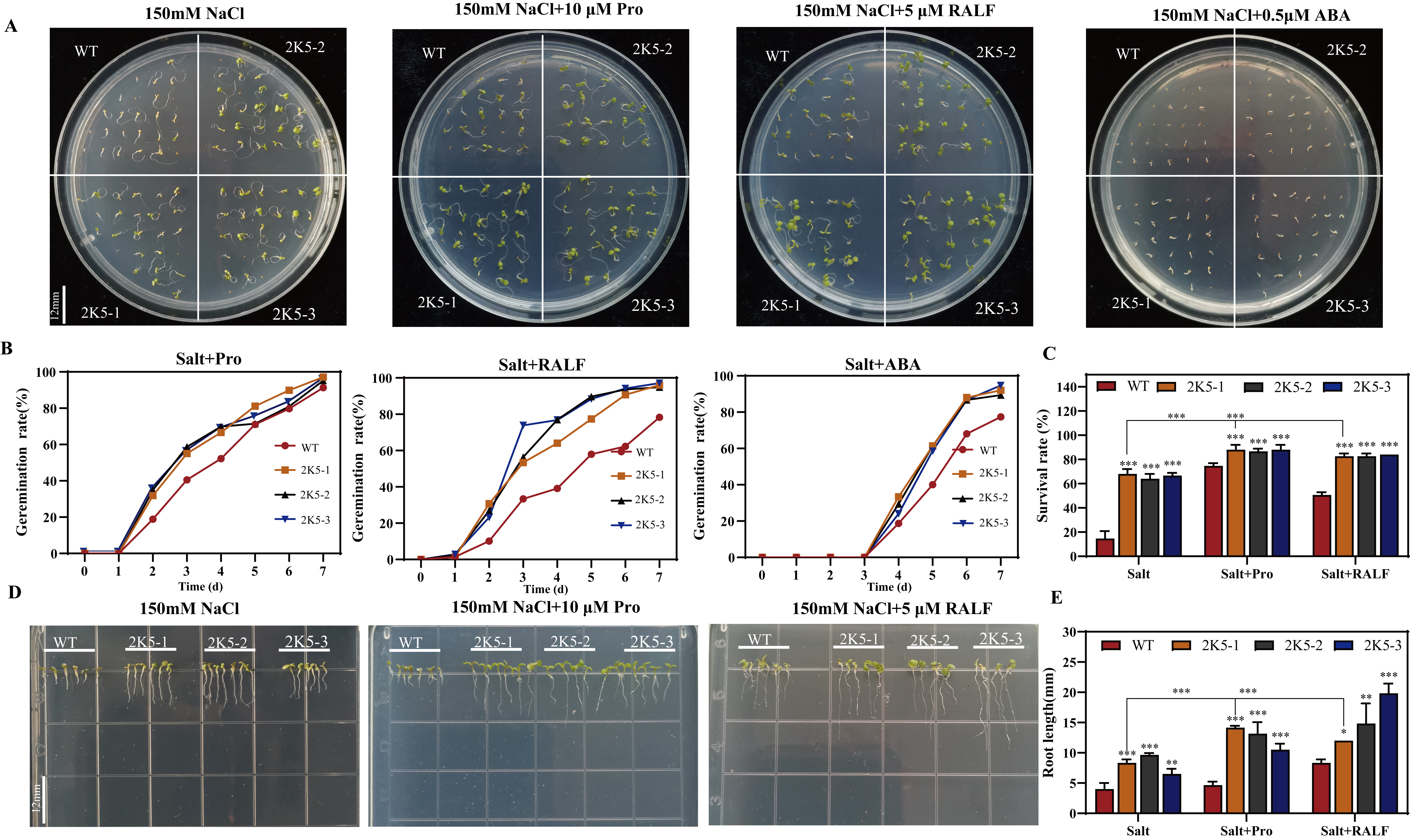


**Supplementary Figure S9** Effects of exogenous substances on transgenic *Arabidopsis thaliana* under salt conditions. (A, B) Germination of seeds under different conditions (C) Survival rate of seeds under different conditions; (D, E) Seedling root growth under different conditions. One-way analysis of variance was used. The error bars represent ± SD of at least three biological replicates (* p < 0.05, ** p< 0.01, *** p < 0.001).


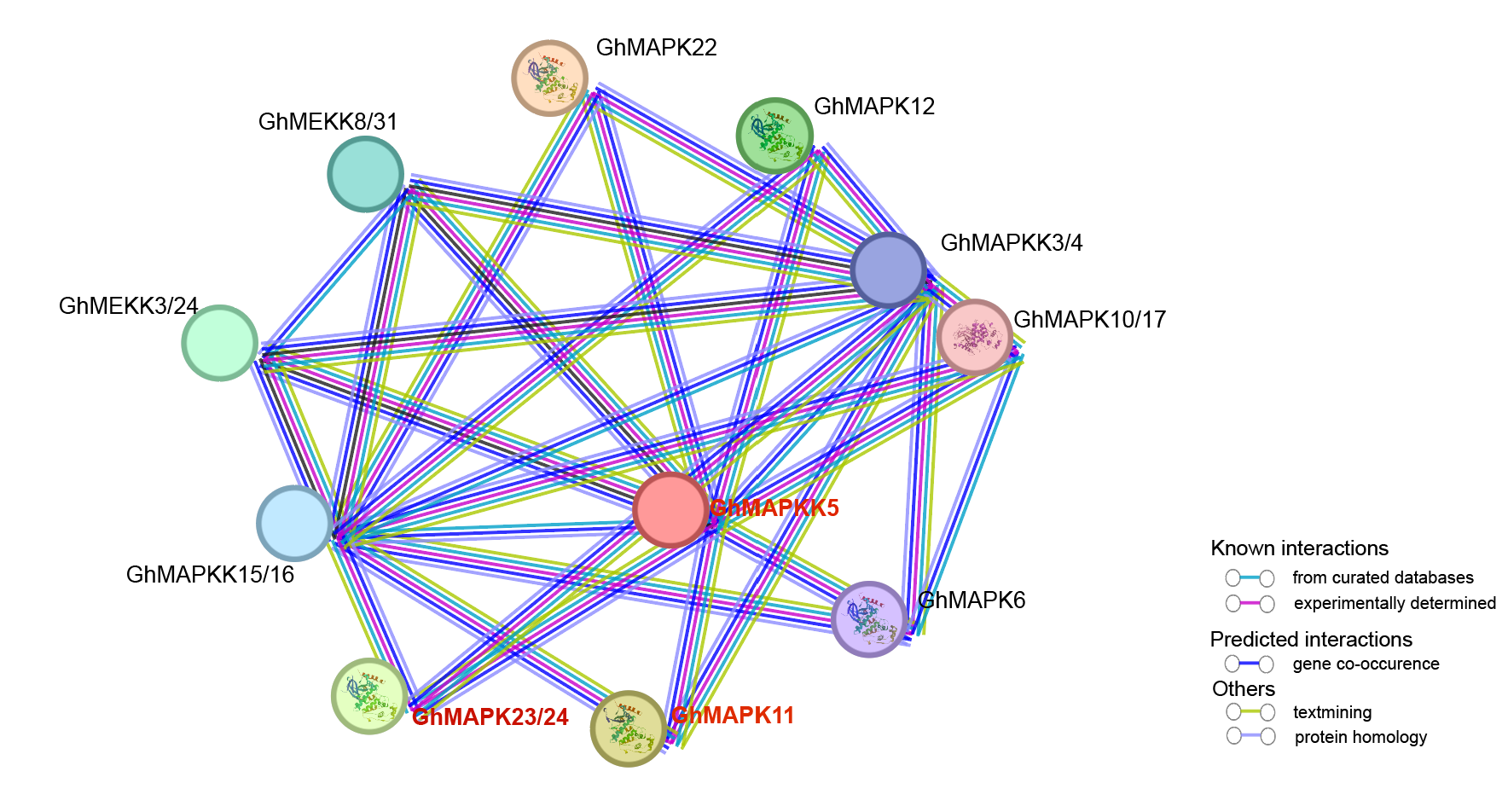


**Supplementary Figure S10** GhMAPKK5 protein interaction prediction map.
