# Supplementary material for: Molecular traits of MAPK kinases and the regulatory mechanism of GhMAPKK5 alleviating drought/salt stress in cotton
Source: Plant Physiol. 2024 Aug 14;196(3):2030–47. doi: 10.1093/plphys/kiae415 (PMC11531841; doi:10.1093/plphys/kiae415)
Supplement: kiae415_Supplementary_Data [file pp.00072.2024-s06.pdf › pp.00072.2024-s06.pdf]

Plant Physiology®

Molecular traits of MAPKK gene family and the regulatory mechanism of GhMAPKK5 alleviating drought/salt stress in cotton

Rui Ding, Junhua Li, Jie Wang, Yan Li, Wuwei Ye, Gentu Yan, and Zujun Yin

Review Timeline:

Submission Date:	30-Jan-2024
Editorial Decision:	29-Feb-2024
Revision Received:	20-May-2024
Editorial Decision:	31-May-2024
Revision Received:	10-Jun-2024
Accepted:	12-Jun-2024

29-Feb-2024

Prof. Zujun Yin
Zhengzhou Research Base, State Key Laboratory of Cotton Biology, School of Agricultural Sciences, Zhengzhou University
institute of cotton research of caas
Zhengzhou
China

RE: Identification of MAPKK gene family and regulatory mechanism of GhMAPKK5 in alleviating drought/salt stress in cotton

Dear Dr. Yin:

We have received reviews of your manuscript, which you may view at the end of this letter. On the basis of the advice received and my own evaluation, I believe that, pending revision, this article may ultimately be acceptable for publication in Plant Physiology.

You will see below that I got two highly positive reviewer comments on your manuscript. In any case, based on the comments of the reviewers, and my reading, you will see that both reviewers have also raised several excellent points that will require your attention and a bit of work before formal acceptance. Although most of the changes required are textual ones you will see that there are significant clarifications of several issues, including (but not limited to):

i) details about your experimental set-up ;
ii) the introduction could be shortened without losing the main message;
iii) the significance of your main results and the main conclusions should be added/modified to your manuscript to provide further support to your data;
iv) several comments were kindly added by reviewer #1 and also raised by reviewer #2 in an attempt to enhance the clarity and logical flow of the manuscript must be followed.

As a note, it seems that reviewer #1 has repeated the comments so just ignore the repetition (just answering once is enough).

I will expect you to bring the manuscript up to the Plant Physiology standard considering you can effectively answer the comments raised and provide an explanation for all queries raised.

Please pay careful attention to the excellent and well-considered points and comments of the reviewers when revising your manuscript which also appears to benefit from extra language attention.

When you are ready to submit your revision, click the below link and find the red arrow under your Author Tasks heading for this revision submission.

If you want to visit this version of the manuscript, you may click the link below and then click on the folder that reads "Post Decision Manuscripts".

Link Not Available

When uploading the revised version of this article, please be sure to include as complete an answer as possible to the referees' criticisms during the first round of reviews in the "response to reviewers" section.

Plant Physiology considers a revision for up to 60 days after a manuscript has been provisionally accepted; limited extension of this timeframe is at the discretion of the Monitoring Editor. A reminder notice will be sent to you 2 weeks before the deadline, at which time you may choose to contact the editorial office to request an extension; if so, please provide a justification for your request. After the expiration of this deadline, the article would need to be submitted as a resubmission and would be handled as such.

Thank you very much for giving us an opportunity to review this work. I look forward to receiving the next version.

Sincerely,

Editor, Plant Physiology

Want to add this revision deadline to your calendar? Click below!

-- Reviewer comments:
Reviewer #1 (Comments for the Author):

This study comprehensively analyzed the MAPKK gene family in 64 plant species. The regulatory mechanism of GhMAPKK5, and the mechanism of MAPK cascade interacting with ROS, ABA, proline and other pathways were well characterized. The results provided empirical evidence of the involvement of MAPKK in alleviating drought and salinity stresses, which provided gene resource and theoretical guidance for the genetic improvement of cotton abiotic stress tolerance. Overall, the experimental design is perfect, the analysis is excellent. The manuscript can be accepted for publication after revision. My major concerns are as follows:

Please pay attention to the use of ABBREVIATION and FULL NAME for some terminologies. The ABBREVATION should be firstly defined at the first mention.
2.Many spelling and typesetting error exist in the current manuscript. Please further check and correct them carefully to make it more readable.
3.INTRODUCTION: The introduction section is too long and verbose. Please revise it to make it more coherent, more readable and more logical.
4.The introduction effectively sets the stage by highlighting the significance of abiotic stresses on crop productivity, specifically mentioning drought and salinity. In particular, the introduction section provides a thorough review of MAPKs' roles in various plant species, which establishes a solid background. However, the section could benefit from a more explicit connection between the reviewed literature and the objectives of this study, and clarify how previous findings directly related to your research on cotton.
5.In materials and methods section, many plant species were analyzed. However, the selection of Tahe-2 as the experimental materials in your study is not sufficiently justified. Given the diversity among cotton varieties, especially in terms of abiotic stress reponses, a more detailed rationale for choosing Tahe-2 is necessary. Please elaborate on any preliminary findings or literature that supports Tahe-2's suitability for studying GhMAPKK5 and its specificity among different cotton varieties. Is there any specificity for GhMAPKK5 and other genes among different cotton varieties？
6.In section 2.3, the description of the experimental setup for the abiotic stress treatments lacks specific details. It is crucial to specify the number of plants used in each treatment and the number of biological replicates. This information is vital for assessing the robustness and reproducibility of your findings. Additionally, clarify the criteria for selecting these numbers to ensure statistical validity.
7.Identification and phylogenetic analysis: The identification of MAPKK gene family members across cotton and other species is clearly laid out. The phylogenetic tree's findings are intriguing, especially the clustering of MAPKK genes into five distinct groups. Providing a more detailed explanation of the significance of these grouping in relation to functional diversification or evolutionary history could enrich the narrative. The observation that MAPKK genes in cotton are more numerous than in many other species is interesting. Expanding on why this might be the case (e.g, specific evolutionary pressures or duplication events unique to cotton) could provide valuable context. Highlighting any implications this might have for cotton breeding for stress resistance could make the results more impactful.
8.The expression of MAPKKs in A.thaliana, O.sativa and G. hirsutum were characterized under different abiotic stress. Do some MAPKK member showed consistent expression pattern changes under multiple abiotic stresses compared with that of the CK? GhMAPKK5 might be one of these like genes. Please supplement the corresponding information if there any other MAPKK members.
9.GhMAPKK5 can improve salt tolerance and drought tolerance. This is a very interesting result. Does allelic variation for GhMAPKK5 exist in cotton germplasm of modern cotton cultivars? Please supplement the corresponding information of the corresponding information is available.
10.A total of 6448 DEGs were overlapped between VIGS vs VIGS_D in comparison to CK vs CK_D, where 3326 DEGs were unique in CK vs CK_D. How many DEGs were unique in VIGS vs VIGS_D. These unique DEGs should be also interesting to the audience including me. Under the absence of the GhMAPKK5, these unique DEGs might account for the reduced abiotic stress tolerance for the VIGS line.
11.The discussion section of your manuscript provides a comprehensive overview of the MAPK cascade's role in plant signal transduction, focusing on the MAPKK gene family in cotton and its implications for abiotic stress tolerance. It would benefit from explicit comparisons between your results and previous findings, particularly where your results confirm, extend, or contradict existing knowledge. Integrating these findings with known signaling pathways and stress response mechanisms could provide a more holistic view of how MAPKK genes contribute to abiotic stress tolerance. Specifically, discussing how these genes interact with other elements of the stress response network in cotton could highlight potential targets for genetic improvement. Where possible, speculate on the functional importance of the observed genetic and expression diversity. Even if definitive functional characterization is beyond the scope of this study, suggesting potential lines of inquiry or hypotheses based on the results would set the stage for future research.
12.There are numerous grammatic/spelling/typesetting errors that need to be corrected. Further, ensuring consistency in

terminology and citation format throughout the manuscript can improve readability. Additionally, simplifying complex sentences and clarifying technical explanations can make the manuscript more accessible to a broader audience.
Please pay attention to the format of reference citation in the main text. Generally, only family name of the first author should be listed in the citation. In addition, the reference should be listed chronologically if more than two references are involved in.
1.Please pay attention to the format of reference citation in the main text. Generally, only family name of the first author should be listed in the citation. In addition, the reference should be listed chronologically if more than two references are involved in.
2.Please pay attention to the use of ABBREVIATION and FULL NAME for some terminologies. The ABBREVATION should be firstly defined at the first mention.
3.Many spelling and typesetting error exist in the current manuscript. Please further check and correct them carefully to make it more readable.
4.INTRODUCTION: The introduction section is too long and verbose. Please revise it to make it more coherent, more readable and more logical.
5.The introduction effectively sets the stage by highlighting the significance of abiotic stresses on crop productivity, specifically mentioning drought and salinity. In particular, the introduction section provides a thorough review of MAPKs' roles in various plant species, which establishes a solid background. However, the section could benefit from a more explicit connection between the reviewed literature and the objectives of this study, and clarify how previous findings directly related to your research on cotton.
6.In materials and methods section, many plant species were analyzed. However, the selection of Tahe-2 as the experimental materials in your study is not sufficiently justified. Given the diversity among cotton varieties, especially in terms of abiotic stress reponses, a more detailed rationale for choosing Tahe-2 is necessary. Please elaborate on any preliminary findings or literature that supports Tahe-2's suitability for studying GhMAPKK5 and its specificity among different cotton varieties. Is there any specificity for GhMAPKK5 and other genes among different cotton varieties？
7.In section 2.3, the description of the experimental setup for the abiotic stress treatments lacks specific details. It is crucial to specify the number of plants used in each treatment and the number of biological replicates. This information is vital for assessing the robustness and reproducibility of your findings. Additionally, clarify the criteria for selecting these numbers to ensure statistical validity.
8.Identification and phylogenetic analysis: The identification of MAPKK gene family members across cotton and other species is clearly laid out. The phylogenetic tree's findings are intriguing, especially the clustering of MAPKK genes into five distinct groups. Providing a more detailed explanation of the significance of these grouping in relation to functional diversification or evolutionary history could enrich the narrative. The observation that MAPKK genes in cotton are more numerous than in many other species is interesting. Expanding on why this might be the case (e.g, specific evolutionary pressures or duplication events unique to cotton) could provide valuable context. Highlighting any implications this might have for cotton breeding for stress resistance could make the results more impactful.
9.The expression of MAPKKs in A.thaliana, O.sativa and G. hirsutum were characterized under different abiotic stress. Do some MAPKK member showed consistent expression pattern changes under multiple abiotic stresses compared with that of the CK? GhMAPKK5 might be one of these like genes. Please supplement the corresponding information if there any other MAPKK members.
10.GhMAPKK5 can improve salt tolerance and drought tolerance. This is a very interesting result. Does allelic variation for GhMAPKK5 exist in cotton germplasm of modern cotton cultivars? Please supplement the corresponding information of the corresponding information is available.
11.A total of 6448 DEGs were overlapped between VIGS vs VIGS_D in comparison to CK vs CK_D, where 3326 DEGs were unique in CK vs CK_D. How many DEGs were unique in VIGS vs VIGS_D. These unique DEGs should be also interesting to the audience including me. Under the absence of the GhMAPKK5, these unique DEGs might account for the reduced abiotic stress tolerance for the VIGS line.
12.The discussion section of your manuscript provides a comprehensive overview of the MAPK cascade's role in plant signal transduction, focusing on the MAPKK gene family in cotton and its implications for abiotic stress tolerance. It would benefit from explicit comparisons between your results and previous findings, particularly where your results confirm, extend, or contradict existing knowledge. Integrating these findings with known signaling pathways and stress response mechanisms could provide a more holistic view of how MAPKK genes contribute to abiotic stress tolerance. Specifically, discussing how these genes interact with other elements of the stress response network in cotton could highlight potential targets for genetic improvement. Where possible, speculate on the functional importance of the observed genetic and expression diversity. Even if definitive functional characterization is beyond the scope of this study, suggesting potential lines of inquiry or hypotheses based on the results would set the stage for future research.
13.While the manuscript is relatively well written, there are numerous grammatic/spelling/typesetting errors that need to be corrected. Further, ensuring consistency in terminology and citation format throughout the manuscript can improve readability. Additionally, simplifying complex sentences and clarifying technical explanations can make the manuscript more accessible to a broader audience.

Reviewer #2 (Comments for the Author):

In the manuscript titled "Identification of MAPKK gene family and the regulatory mechanism of GhMAPKK5 in alleviating drought/salt stress in cotton", Ding and colleagues conducted a thorough analysis of the MAPKK gene family across various plant species. They also performed in silico analysis, demonstrating that GhMAPKK5 contributes to salt and drought tolerance in

cotton, supported by gain-of function and loss-of-function studies in Arabidopsis and cotton, respectively. Global transcriptomics analysis suggests that GhMAPKK5 likely functions upstream of GhMAPK23/GhMAPK24 at the transcriptional level, playing a key role in the regulation of ABA, proline, and small peptides under stress conditions. While the MAPKK is a key component in the MAPK signaling cascades in Arabidopsis, its role in other plant species, such as cotton, remains poorly understood. This manuscript elaborated on how GhMAPKK5 may work in cotton, offering new avenue to enhance stress tolerance in this crop. However, I have pointed out several places that need to be amended before being accepted for publication.

1.In the introduction section, the phrase "NaCl-induced via activation of MPK6 and transmits upstream signals to MPK3/4, mediating compatible pollination (Jamshed et al., 2020)" should be revised for clarity and consistency. Specifically, MPK6 and MPK3/4 should be annotated as AtMPK6 and AtMPK3/4, respectively, to align with the naming convention used for other AtMAPKs throughout the manuscript. The same problem appears on lines 110, 111, and more. In addition, mitogen-activated protein kinase is referred to as MAPKK, MKK, MEK. There are three forms of abbreviation appear, which can be used in a more unified way.
2.Line 151-153 Add the web address of database.
3.I understood that the last paragraph of Materials and methods defined error bars' meaning in all figures and clarified statistically analysis. However, it would enhance clarity to repeat this information in the figure legends, particularly for Figures 6 and 7 where the definition of error bars is included, but it is missing in Figures like 5C, 4D, and 11C-F.
4.Line 283-284, 100 mM ABA, 100 μM proline and 10 μM RALF solution was sprayed. Why chose these concentration? The experimental method was not detailed enough. What tissue is treated with ABA, proline and RALF solution? How to spray? After treatments, plants were imaged and harvested to analyze serial physiological parameters" when?
5.The Methods section would benefit from a more detailed descriptions of the RT-PCR protocol, especially regarding primer design. Including specific information on how the primers were chosen and how they target the relevant transcripts would provide valuable insight into the assay's specificity and reliability.

6.In the results section, a total of 478 MAPKK genes in 64 plant species were recognized. The first paragraph should provide an concise summary of the identification and characterization analysis of the MAPKK gene family across the plant kingdom. Following this overview, a more detailed analysis of the MAPKK gene family in both diploid and tetraploid cotton should be presented. Given the Gossypium genus' suitability for studying the effects of polyploidy, an evolutionary perspective on the MAPKK genes in diploid and tetraploid cotton would provide valuable insights into how polyploidy contributes to genomic sequence variation and evolutionary innovation.

7.Line 676-679 The number of MAPKK genes (11 to 26) in cotton were much higher than many other plant species, such as 5 in Vitis vinifera, 8 in Picea abies, and 8 in O. sativa, but there was no correlation between genome size and the number of MAPKK genes. Discuss that other than repeat the results.

8.Line 436-439 Rewrite this sentence "The expression of MAPKKs in A.thaliana, O.sativa and G. hirsutum after cold, heat, salt, and PEG stresses and verified them by qRT-PCR using existing RNA-seq data were analyzed in order to further understand the response of MAPKK genes to abiotic stress. A" . The expression of MAPKKs in O.sativa was not analyzed in this part.

9.Regarding the structure of the Results section, the use of a phylogenetic tree for the evolutional analysis of MAPKK families, coupled with expression analysis, effectively identifies GhMAPKK5 as a strong candidate gene for stress tolerance in cotton. However, the sequence of experimental validation described -mentioning overexpression work in Arabidopsis before VIGS in cotton -could be optimized for logical flow. It is recommended to first present the VIGS work in cotton to validate the gene's function with its native context, followed by the overexpression studies in Arabidopsis. This order emphasizes the gene's role in cotton before exploring its potential application or function in other plant species.

10.The mitigating effects of exogenous ABA, proline, and RALF on the stress sensitivity of GhMAPKK5-silenced cotton, as supported by RNA-seq analysis, present an interesting aspect of stress physiology. The subsequent improvement in stress tolerance of Arabidopsis plants through GhMAPKK5 overexpression raises questions about the mechanisms at play. It would be beneficial to explore how ABA, proline, and RALF signaling pathways interact with GhMAPKK5 overexpression in transgenic Arabidopsis. Does GhMAPKK5 overexpression alter the expression of these elements in Arabidopsis plants?

11.To ensure consistency across the manuscript, the wording of figure legends regarding statistical information should be standardized. For example, the legend for Fig. 10 could be revised to match the format used in Fig. 6, such as 'The error bars represent {plus minus} SD of at least three biological replicates (* p < 0.05, ** p < 0.01, *** p < 0.001).' This adjustment will provide clarity and uniformity in how statistical data are presented throughout the manuscript.

12.As author claimed that GhMAPKK5 may function via phosphorylation of GhMAPK23/24 to confers cotton response to stress. In the MAPK cascade, whether the upstream and downstream genes of GhMAPKK5 can be determined by relevant experiments. For example, the interaction between GhMAPK23/24 and GhMAPKK5 has been confirmed based on existing data. In addition, based on protein interaction predictions illustrated in the accompanying figure, proteins such as GhMEKK3/8/31/24, are potential interactors with GhMAPKK5. It is therefore suggested that verification experiments be conducted to elucidate a more comprehensive MAPK cascade pathway. Demonstrating this interaction would strengthen the proposed signaling pathway and provide a clearer understanding of the molecular dynamics at play.

13.For Fig. 11C-F, it is important to specify the number of biological replicates used to generate the data.

14. Identifying the closet AtMAPKK homolog(s) to GhMAPKK5 in cotton and discussing the extent of research conducted on these Arabidopsis AtMAKK(s) would enrich the discussion. This comparison could shed light on potential functional similarities or differences between the MAPKKs of these two species and offer a broader evolutionary perspective. If space permits, including this analysis in the discussion would provide a valuable context for understanding the significance of GhMAPKK's role in stress tolerance.

15.Line 714-716, Line 724-725 Delete these sentences because they are well-known. Line 713-748 Simplify this part and find the common expression trend of MAPKKs in different species under the same treatments. Line 749-775 Rewrite these sentences in discussion manner. Line 775-790 Delete.

16.While the research content is commendable, improvements in the manuscript's writing and organization are necessary to enhance its clarity and logical flow. Streaming the presentation of results, refining the language for coherence, and ensuring a logical progression of ideas will significantly improve the manuscript's overall quality.

Dear Reviewer,

We are very grateful for your comments and thoughtful suggestions. In the revision, we have made more comprehensive modifications to address your comments and concerns. Based on the above views, we added experiments such as yeast two-hybrid verification of GhMAPKK5 interacting proteins, exogenous substances applied to overexpression of Arabidopsis and detection of the expression levels of related pathways before and after overexpression of Arabidopsis under drought and salt stress to further study the regulatory pathways involved in *GhMAPKK5*. These experiments make our results richer and more convincing. In addition to this, we have also made changes to the introduction and discussion sections to make them more readable. Below are our responses to the suggestions in an item-by-item manner.

Reviewer#1:

1. Please pay attention to the use of ABBREVIATION and FULL NAME for some terminologies. The ABBREVATION should be firstly defined at the first mention.

Response: Thank you for your suggestion. We have added information to the relevant abbreviations.

2. Many spelling and typesetting error exist in the current manuscript. Please further check and correct them carefully to make it more readable.

Response: Thank you. We checked it carefully and made changes.

3. Introduction: The introduction section is too long and verbose. Please revise it to make it more coherent, more readable and more logical. And 4. the introduction effectively sets the stage by highlighting the significance of abiotic stresses on crop productivity, specifically mentioning drought and salinity. In particular, the introduction section provides a thorough review of MAPKs' roles in various plant species, which establishes a solid background. However, the section could benefit from a more explicit connection between the reviewed literature and the objectives of this study, and clarify how previous findings directly related to your research on cotton.

Response: Thank you. We have summarized and modified the content of the introduction.

5. In materials and methods section, many plant species were analyzed. However, the selection of Tahe-2 as the experimental materials in your study is not sufficiently justified. Given the diversity among cotton varieties, especially in terms of abiotic stress response, a more detailed rationale for choosing Tahe-2 is necessary. Please elaborate on any preliminary findings or literature that supports Tahe-2's suitability for studying *GhMAPKK5* and its specificity among different cotton varieties. Is there any specificity for *GhMAPKK5* and other genes among different cotton varieties？

Response: Thank you for your suggestion. The direct expression of *GhMAPKK5* was specific in different varieties. According to the previous study (Yuan et al., 2023), we carried out drought and salt treatment on 12 cotton varieties, including TM-1, KF868, TaHe-2, XQ-1, Han5158, CCRI9001, YS6, LMY16, Han117, LM6, CCRI12, and CCRI9001, and sampled cotton leaves before and after stress treatment (Lines 216-218, page 11).

The expression of *GhMAPKK5* in Tahe-2 and drought-tolerant and salt-tolerant varieties CCRI9001 and CCRI49, was detected by fluorescence quantification, and the expression of *GhMAPKK5* was slightly higher than that in other varieties. The expression of *GhMAPKK5* in Tahe-2 was the highest, so Tahe-2 was chosen as the research material (Lines 401-404, page 19).

6. In section 2.3, the description of the experimental setup for the abiotic stress treatments lacks

specific details. It is crucial to specify the number of plants used in each treatment and the number of biological replicates. This information is vital for assessing the robustness and reproducibility of your findings. Additionally, clarify the criteria for selecting these numbers to ensure statistical validity.

Response: Thank you for your suggestion. 45 seedlings with similar growth were used for each treatment. 15 seedlings were used in each replicate. This information has been provided in the revised "Materials and methods" (Line188-189 page 9).

7. Identification and phylogenetic analysis: The identification of *MAPKK* gene family members across cotton and other species is clearly laid out. The phylogenetic tree's findings are intriguing, especially the clustering of *MAPKK* genes into five distinct groups. Providing a more detailed explanation of the significance of these grouping in relation to functional diversification or evolutionary history could enrich the narrative. The observation that *MAPKK* genes in cotton are more numerous than in many other species is interesting. Expanding on why this might be the case (e.g, specific evolutionary pressures or duplication events unique to cotton) could provide valuable context. Highlighting any implications this might have for cotton breeding for stress resistance could make the results more impactful.

Response: Thank you for your suggestion. Through the analysis of gene structure and conserved motif in different groups, it was found that most of the genes in group E belonged to PK superfamily, which also reflected the functional diversity in evolution, which was supplemented in the discussion section. (Line 589-593 page 28)

According to collinearity and *Ka*/*Ks* analysis, the number of *MAPKK* genes in cotton is mainly due to whole-genome replication and fragment repetition, and is relatively conserved in evolution. (Line 323-333 page 16).

8. The expression of *MAPKKs* in *A.thaliana*, *O.sativa* and *G. hirsutum* were characterized under different abiotic stress. Do some *MAPKK* member showed consistent expression pattern changes under multiple abiotic stresses compared with that of the CK? *GhMAPKK5* might be one of these like genes. Please supplement the corresponding information if there any other *MAPKK* members.

Response: Thank you for your suggestion. Yes, different subgroups of genes often exhibit similar expression patterns under different abiotic stress treatments. In the analysis of expression patterns, we classified the genes of the same subgroup and analyzed them uniformly (Line393-396 page 19).

9. *GhMAPKK5* can improve salt tolerance and drought tolerance. This is a very interesting result. Does allelic variation for GhMAPKK5 exist in cotton germplasm of modern cotton cultivars? Please supplement the corresponding information of the corresponding information is available.

Response: Thank you for your suggestion. With regard to the existence of *GhMAPKK5* allele variation in the germplasm of modern cotton varieties, we found that there was no research on the existence of *GhMAPKK5* allele variation in the germplasm of modern cotton varieties.

10. A total of 6448 DEGs were overlapped between VIGS vs VIGS_D in comparison to CK vs CK_D, where 3326 DEGs were unique in CK vs CK_D. How many DEGs were unique in VIGS vs VIGS_D. These unique DEGs should be also interesting to the audience including me. Under the absence of the GhMAPKK5, these unique DEGs might account for the reduced abiotic stress tolerance for the VIGS line.

Response: Thank you for your suggestion. Compared to CK VS CK_D, 6168 DEGs are unique in VIGS and VIGS_D. There were a large number of these genes. Subsequent KEGG analysis and GO analysis of these differentially expressed genes found that they were mostly concentrated in

serine/tryptophan kinase activity and protein phosphorylation, and the main enrichment pathway was in the MAPK signaling pathway. (Line437-440 page 21; Line456-460 page 22)

11. The discussion section of your manuscript provides a comprehensive overview of the MAPK cascade's role in plant signal transduction, focusing on the *MAPKK* gene family in cotton and its implications for abiotic stress tolerance. It would benefit from explicit comparisons between your results and previous findings, particularly where your results confirm, extend, or contradict existing knowledge. Integrating these findings with known signaling pathways and stress response mechanisms could provide a more holistic view of how *MAPKK* genes contribute to abiotic stress tolerance. Specifically, discussing how these genes interact with other elements of the stress response network in cotton could highlight potential targets for genetic improvement. Where possible, speculate on the functional importance of the observed genetic and expression diversity. Even if definitive functional characterization is beyond the scope of this study, suggesting potential lines of inquiry or hypotheses based on the results would set the stage for future research.

Response: Thank you for your suggestion. We have modified the contents of the discussion section based on your suggestions.

12. There are numerous grammatic/spelling/typesetting errors that need to be corrected. Further, ensuring consistency in terminology and citation format throughout the manuscript can improve readability. Additionally, simplifying complex sentences and clarifying technical explanations can make the manuscript more accessible to a broader audience.

Response: Thank you for your suggestion. We have modified the contents of the discussion section based on your suggestions. According to the suggestion, we unify the writing methods of *MAPKK* and *MAP2K* in the article and write them as *MAPKK*. And found a number of spelling errors, have been corrected.

Reviewer#2:

1. In the introduction section, the phrase "NaCl-induced via activation of MPK6 and transmits upstream signals to MPK3/4, mediating compatible pollination (Jamshed et al., 2020)" should be revised for clarity and consistency. Specifically, MPK6 and MPK3/4 should be annotated as AtMPK6 and AtMPK3/4, respectively, to align with the naming convention used for other AtMAPKs throughout the manuscript. The same problem appears on lines 110, 111, and more. In addition, mitogen-activated protein kinase is referred to as MAPKK, MKK, MEK. There are three forms of abbreviation appear, which can be used in a more unified way.

Response: Thank you for your suggestion. Harmonization and modification of gene names have been carried out. According to the suggestion, we unify the writing methods of *MAPKK* and *MAP2K* in the article and write them as *MAPKK*. (Line59-71 page 4)

2. Line 151-153 Add the web address of database.

Response: Thank you. We added the database information. (Line105-107 page 6)

3. I understood that the last paragraph of Materials and methods defined error bars' meaning in all figures and clarified statistically analysis. However, it would enhance clarity to repeat this information in the figure legends, particularly for Figures 6 and 7 where the definition of error bars is included, but it is missing in Figures like 5C, 4D, and 11C-F.

Response: Thank you for your suggestion. We carefully reviewed and added the definition of error bars as well as statistical analysis to the legend in Figure 4, Figure 5, Figure 6, Figure 7, Figure 8 and Supplementary Figure 4, and 9.

4. Line 283-284, 100 mM ABA, 100 μM proline and 10 μM RALF solution was sprayed. Why chose these concentrations? The experimental method was not detailed enough. What tissue is treated with ABA, proline and RALF solution? How to spray? After treatments, plants were imaged and harvested to analyze serial physiological parameters" when?

Response: Thank you for your suggestion. The selection of the concentration of foreign substances used in the experiment was determined through literature review (胡阳光, 2019; 秦可臻, 2019; 沙汉景, 2013; 王小文, 2023) and pre-experiment. Cotton leaves appeared wilting after 6 h of treatment with these concentrations. It was treated by spraying evenly on the blades, and samples were taken after 6 h of treatment. The addition of foreign substances in Arabidopsis thaliana was determined by pre-experiment. This part has been added to the materials and methods. (Line241-249 page 12; Line268-271 page 13)

5. The Methods section would benefit from a more detailed descriptions of the RT-PCR protocol, especially regarding primer design. Including specific information on how the primers were chosen and how they target the relevant transcripts would provide valuable insight into the assay's specificity and reliability.

Response: Thank you for your suggestion. Primers were designed using online tools in genscript website (https://www.genscript.com/tools/real-time-pcr-taqman-primer-design-tool) according to primer design principles, and the specificity of primers was detected by prime-blast in NCBI (https://www.ncbi.nlm.nih.gov/tools/primer-blast/index.cgi). Before the experiment, RT-PCR and agarose gel electrophoresis were performed to verify the specificity of the primers. We have added this part to the material method. (Line208-212 page 10)

6. In the results section, a total of 478 *MAPKK* genes in 64 plant species were recognized. The first paragraph should provide a concise summary of the identification and characterization analysis of the *MAPKK* gene family across the plant kingdom. Following this overview, a more detailed analysis of the *MAPKK* gene family in both diploid and tetraploid cotton should be presented. Given the Gossypium genus' suitability for studying the effects of polyploidy, an evolutionary perspective on the *MAPKK* genes in diploid and tetraploid cotton would provide valuable insights into how polyploidy contributes to genomic sequence variation and evolutionary innovation.

Response: Thank you. We adjusted the content of the first section of the result. (Line298-319 page 15)

7. Line 676-679 The number of *MAPKK* genes (11 to 26) in cotton were much higher than many other plant species, such as 5 in Vitis vinifera, 8 in Picea abies, and 8 in O. sativa, but there was no correlation between genome size and the number of *MAPKK* genes. Discuss that other than repeat the results.

Response: Thank you for your suggestion. We put this in the first section of the results, with examples to prove it. (Line302-305 page 15)

8. Line 436-439 Rewrite this sentence "The expression of *MAPKKs* in *A.thaliana*, *O.sativa* and *G. hirsutum* after cold, heat, salt, and PEG stresses and verified them by qRT-PCR using existing RNA-seq data were analyzed in order to further understand the response of *MAPKK* genes to abiotic stress. A". The expression of *MAPKKs* in O.sativa was not analyzed in this part.

Response: Thank you for your suggestion. We added the analysis of *MAPKK* gene expression pattern in rice to the results. (Line368-371 page 18; Line 389-391 page 19)

9. Regarding the structure of the Results section, the use of a phylogenetic tree for the evolutional

analysis of *MAPKK* families, coupled with expression analysis, effectively identifies *GhMAPKK5* as a strong candidate gene for stress tolerance in cotton. However, the sequence of experimental validation described-mentioning overexpression work in Arabidopsis before VIGS in cotton - could be optimized for logical flow. It is recommended to first present the VIGS work in cotton to validate the gene's function with its native context, followed by the overexpression studies in Arabidopsis. This order emphasizes the gene's role in cotton before exploring its potential application or function in other plant species.

Response: Thank you for your suggestion. The results of the paper were modified according to the suggestions, and the overexpression of Arabidopsis was placed after the VIGS verification function.

10. The mitigating effects of exogenous ABA, proline, and RALF on the stress sensitivity of *GhMAPKK5*-silenced cotton, as supported by RNA-seq analysis, present an interesting aspect of stress physiology. The subsequent improvement in stress tolerance of Arabidopsis plants through *GhMAPKK5* overexpression raises questions about the mechanisms at play. It would be beneficial to explore how ABA, proline, and RALF signaling pathways interact with *GhMAPKK5* overexpression in transgenic Arabidopsis. Does *GhMAPKK5* overexpression alter the expression of these elements in Arabidopsis plants?

Response: Thank you for your suggestion. In transgenic Arabidopsis, exogenous substances ABA, proline and RALF were studied on the germination rate, survival rate and root growth of overexpressed Arabidopsis seeds to prove the influence of exogenous substances. Moreover, through the detection of the expression levels of related genes such as *FER*, *PYL* and *SnRK*, it was proved that *GhMAPKK5* could indeed affect ABA, ROS, proline and RALF-related pathways. (Line535-540 page 26; Figure 8 G, Supplementary Figure 9 and 10)

11. To ensure consistency across the manuscript, the wording of figure legends regarding statistical information should be standardized. For example, the legend for Fig. 10 could be revised to match the format used in Fig. 6, such as 'The error bars represent SD of at least three biological replicates (* $p < 0.05$, ** $p < 0.01$, *** $p < 0.001$).' This adjustment will provide clarity and uniformity in how statistical data are presented throughout the manuscript.

Response: Thank you for your suggestion. We carefully reviewed and added the definition of error bars as well as statistical analysis to the legend in Figure 4, Figure 5, Figure 6, Figure 7, Figure 8 and Supplementary Figure 4, 9 and 10.

12. As author claimed that *GhMAPKK5* may function via phosphorylation of *GhMAPK23/24* to confers cotton response to stress. In the MAPK cascade, whether the upstream and downstream genes of *GhMAPKK5* can be determined by relevant experiments. For example, the interaction between *GhMAPK23/24* and *GhMAPKK5* has been confirmed based on existing data. In addition, based on protein interaction predictions illustrated in the accompanying figure, proteins such as *GhMEKK3/8/31/24*, are potential interactors with *GhMAPKK5*. It is therefore suggested that verification experiments be conducted to elucidate a more comprehensive MAPK cascade pathway. Demonstrating this interaction would strengthen the proposed signaling pathway and provide a clearer understanding of the molecular dynamics at play.

Response: Thank you for your suggestion. In order to further validate the proteins interacting with GhMAPKK5, we conducted a yeast two-hybrid experiment, which has been supplemented with material methods. (Line272-284 page 14)

Through Y2H analysis, it is concluded that GhMEKK3, GhMEKK8, GhMEKK14, GhMEKK31,

GhMAPK11 and GhMAPK23 can interact with GhMAPKK5, and the interaction network diagram is drawn to obtain a complete MAPK cascade module: GhMEKK3/GhMEKK8/GhMEKK31-GhMAPKK5-GhMAPK11/23 (Line555-570 page 27; Figure 9).

13.For Fig. 11C-F, it is important to specify the number of biological replicates used to generate the data.

Response: Thank you for your suggestion. 45 seedlings with similar growth were used for each treatment. 15 seedlings were used in each replicate. This information has been provided in the revised "Materials and methods" (Line185-186 page 9).

14. Identifying the closet *AtMAPKK* homolog(s) to *GhMAPKK5* in cotton and discussing the extent of research conducted on these Arabidopsis *AtMAKK*(s) would enrich the discussion. This comparison could shed light on potential functional similarities or differences between the *MAPKKs* of these two species and offer a broader evolutionary perspective. If space permits, including this analysis in the discussion would provide a valuable context for understanding the significance of *GhMAPKK*5 role in stress tolerance.

Response: Thank you for your suggestion. In Arabidopsis, *AtMAPKK2* is a homologous gene of *GhMAPKK5*, and the drought-tolerant and salt-tolerant function of *AtMAPKK2* is added to the discussion section. (Line 600-607 page 29).

15.Line 714-716, Line 724-725 Delete these sentences because they are well-known. Line 713-748 Simplify this part and find the common expression trend of *MAPKKs* in different species under the same treatments. Line 749-775 Rewrite these sentences in discussion manner. Line 775-790 Delete.

Response: Thank you for your suggestion. They have been changed.

16.While the research content is commendable, improvements in the manuscript's writing and organization are necessary to enhance its clarity and logical flow. Streaming the presentation of results, refining the language for coherence, and ensuring a logical progression of ideas will significantly improve the manuscript's overall quality.

Response: Thank you for your suggestion. We have made improvements in writing and language organization

Yuan, Z., Zhang, C., Zhu, W., Yan, G., Chen, X., Qiu, P., Ruzimurod, B., Ye, W., Qaraevna, B. Z., & Yin, Z. (2023). Molecular mechanism that underlies cotton response to salt and drought stress revealed by complementary transcriptomic and iTRAQ analyses. Environmental and Experimental Botany, 209. https://doi.org/10.1016/j.envexpbot.2023.105288

胡阳光. (2019). 棉花 ALDH7 和 ALDH1 基因在盐和干旱胁迫下功能初步分析. [河南大学]. 河南.

秦可臻. (2019). RALF 信号调控番茄生长发育和低温抗性的功能研究. [浙江大学]. 浙江.

沙汉景. (2013). 外源脯氨酸对盐胁迫下水稻耐盐性的影响. [东北农业大学]. 黑龙江.

王小文. (2023). GhNAC1 调控棉花耐盐性的机理研究. [山东师范大学]. 山东.

31-May-2024

Prof. Zujun Yin
Zhengzhou Research Base, State Key Laboratory of Cotton Bio-breeding and Integrated Utilization, School of Agricultural Sciences, Zhengzhou University; State Key Laboratory of Cotton Bio-breeding and Integrated Utilization, Institute of Cotton Research, Chinese Academy of Agricultural Sciences; Xinjiang Tarim River Seed Industry Co., Ltd.
institute of cotton research of caas
Anyang
China

RE: Molecular traits of MAPKK gene family and the regulatory mechanism of GhMAPKK5 alleviating drought/salt stress in cotton

Dear Dr. Zujun Yin:

We are pleased to accept your manuscript for publication in the Plant Physiology. This acceptance is contingent on revision based on the minor comments of our reviewers. In particular, please consider the following:

Please pay careful attention to the excellent and well-considered points from reviewer #2 when revising your manuscript which also appears to benefit from extra language attention.

Please highlight all changes and include a detailed annotation to changes to the text, with line numbers, and noting your responses to the comments.

To submit your revised manuscript, click:

Link Not Available

If you cannot return the revised manuscript within 8 weeks of receipt, please let us know. Otherwise, we will assume that you have elected not to revise the manuscript and are withdrawing it.

Thank you for allowing us to review your work. We look forward to hearing from you soon.

Sincerely,

Editor, Plant Physiology

-- Reviewer comments:
Reviewer #1 (Comments for the Author):

All comments and suggestions have been well addressed. The manuscript in the current version can be considred for publish in Plant Physiologhy.

Reviewer #2 (Comments for the Author):

The revised manuscript "Molecular traits of MAPKK gene family and the regulatory mechanism of GhMAPKK5 alleviating drought/salt stress in cotton" has undergone comprehensive revisions and made a significant improvement. After addressing a few minor issues, it can be acceptable.

1. Lines 63-66, "AtMAPKK3....... and coupling with AtMAPKKK18.... ". MAPKK should be activated by MAP3K phosphorylation in the MAPK cascade. The description is not accurate enough in this sentence.
2. Lines 402-403，the last sentence is repeated in the Materials and Methods section. Recommended to remove.
3. In lines 476, in the sentence "...... and (Proline Transporter (ProT) families, ... " There is a parenthesis redundancy.
4. In lines 672-674, "plant BA receptors known as PYR/PYL/RCAR was significantly......", maybe "Plant ABA ......".
4. Authors should read through the manuscript and make sure accurate English expression.

Dear Reviewer,

We are very grateful for your comments and thoughtful suggestions. In the revision, we have made modifications to address your comments and concerns. Below are our responses to the suggestions in an item-by-item manner.

Reviewer#1:

1. Lines 63-66, "AtMAPKK3....... and coupling with AtMAPKKK18.... ". MAPKK should be activated by MAP3K phosphorylation in the MAPK cascade. The description is not accurate enough in this sentence.

Response: Thank you for your suggestion. In lines 65, they have been changed.

2. Lines 402-403， the last sentence is repeated in the Materials and Methods section. Recommended to remove.

Response: Thank you for your suggestion. This sentence has been deleted.

3. In lines 476, in the sentence "...... and (Proline Transporter (ProT) families, ... " There is a parenthesis redundancy.

Response: Thank you for your suggestion. They have been changed.

4. In lines 672-674, "plant BA receptors known as PYR/PYL/RCAR was significantly......", maybe "Plant ABA ......".

Response: Thank you for your suggestion. They have been changed.

5. Authors should read through the manuscript and make sure accurate English expression.

Response: Thank you for your suggestion. We have made improvements in writing and language organization.

12-Jun-2024
Prof. Zujun Yin
Zhengzhou Research Base, State Key Laboratory of Cotton Bio-breeding and Integrated Utilization, School of Agricultural Sciences, Zhengzhou University; State Key Laboratory of Cotton Bio-breeding and Integrated Utilization, Institute of Cotton Research, Chinese Academy of Agricultural Sciences; Xinjiang Tarim River Seed Industry Co., Ltd.
institute of cotton research of caas
Anyang
China

MSID: PP2024-RA-00072R2
MS TITLE: Molecular traits of MAPKK gene family and the regulatory mechanism of GhMAPKK5 alleviating drought/salt stress in cotton

Dear Dr. Zujun Yin:

I am pleased to inform you that your revised manuscript "Molecular traits of MAPKK gene family and the regulatory mechanism of GhMAPKK5 alleviating drought/salt stress in cotton" has been accepted for publication in Plant Physiology. Your manuscript will be checked for consistency with journal formatting and image standards by one of our Science Editors, who will contact you shortly. They may have a number of queries and suggestions for you. Please keep in mind that they work to increase the online visibility of your article and its attraction for readers from the widest of backgrounds. You will have the opportunity to review any revisions, and I hope you will work constructively with them to get the most out of your research. You will also have the opportunity to review all changes when you receive the author proofs for your article.

Once validated for publication, your article will be sent to a Science Editor for a final review of your manuscript and source files. After your final files are uploaded, your article will appear in the next available issue and the online Preview version of your article will be posted after your signed Copyright and Checklist forms and final source files are received.

Please be sure to include the following in your manuscript file:
Harvard style citations
List of all author contributions and funding information at the beginning of manuscript after the titles and author list.
List all supplemental materials associated with the article, with titles, at the end of the ms, before the references and acknowledgments section.
Complete Figure Legends for all figures appearing in the article listed before the Literature Cited.

Thank you and congratulations on your Plant Physiology paper!

Sincerely,
Editor, Plant Physiology

=====
FIRST AUTHOR PROFILES
=====

We recognize the hard work that goes into being First Author of a Plant Physiology paper by publishing a "First Author Feature" for each First Author alongside your paper.

If the first author(s) would like to submit information for a feature on Facebook and Twitter, fill out the form by clicking on the link below:

Plant Physiology First Author Feature Form: https://forms.office.com/r/ZBCGZfhm8F
If you cannot access the form through the link above, please contact Rachel at rbelsky@aspb.org.

To upload an image of yourself to accompany your profile, please put your photo in the correct journal folder at the link below. You must label your image: Author First Name Last Name Article Title.

First Author Profile Image Dropbox: https://www.dropbox.com/scl/fo/xz029p7qjr2jml2rp2kmq/h?dl=0&rlkey=6fkxcaqmxx35ap152b2osy1fz

=====
IMPORTANT REMINDER: PEER REVIEW REPORTS
=====

If you opted to publish a peer review report along with your article during the original submission process, it will be prepared by the editorial staff and publicly posted with your manuscript, inside the zip file that contains any other supplemental material. As a reminder, the peer review report is a public record of all comments from editors and reviewers, as well as your prior responses, as you received them in the decision letters for each draft of your manuscript. If you agreed to publish this report and have changed your mind, or are not sure if you selected this option, please contact the editorial office as soon as possible before signing the license agreement from our publisher.

-- Reviewer comments:
